# Formation and persistence of polyglutamine aggregates in mistranslating cells

**DOI:** 10.1093/nar/gkab898

**Published:** 2021-10-28

**Authors:** Jeremy T Lant, Rashmi Kiri, Martin L Duennwald, Patrick O’Donoghue

**Affiliations:** Department of Biochemistry, The University of Western Ontario, London, Ontario N6A 5C1, Canada; Department of Biochemistry, The University of Western Ontario, London, Ontario N6A 5C1, Canada; Department of Anatomy & Cell Biology, The University of Western Ontario, London, Ontario N6A 5C1, Canada; Department of Biochemistry, The University of Western Ontario, London, Ontario N6A 5C1, Canada; Department of Chemistry, The University of Western Ontario, London, Ontario N6A 5C1, Canada

## Abstract

In neurodegenerative diseases, including pathologies with well-known causative alleles, genetic factors that modify severity or age of onset are not entirely understood. We recently documented the unexpected prevalence of transfer RNA (tRNA) mutants in the human population, including variants that cause amino acid mis-incorporation. We hypothesized that a mistranslating tRNA will exacerbate toxicity and modify the molecular pathology of Huntington's disease-causing alleles. We characterized a tRNA^Pro^ mutant that mistranslates proline codons with alanine, and tRNA^Ser^ mutants, including a tRNA^Ser^_AGA_ G35A variant with a phenylalanine anticodon (tRNA^Ser^_AAA_) found in ∼2% of the population. The tRNA^Pro^ mutant caused synthetic toxicity with a deleterious huntingtin poly-glutamine (polyQ) allele in neuronal cells. The tRNA^Ser^_AAA_ variant showed synthetic toxicity with proteasome inhibition but did not enhance toxicity of the huntingtin allele. Cells mistranslating phenylalanine or proline codons with serine had significantly reduced rates of protein synthesis. Mistranslating cells were slow but effective in forming insoluble polyQ aggregates, defective in protein and aggregate degradation, and resistant to the neuroprotective integrated stress response inhibitor (ISRIB). Our findings identify mistranslating tRNA variants as genetic factors that slow protein aggregation kinetics, inhibit aggregate clearance, and increase drug resistance in cellular models of neurodegenerative disease.

## Graphical Abstract

**Graphical Abstract:**
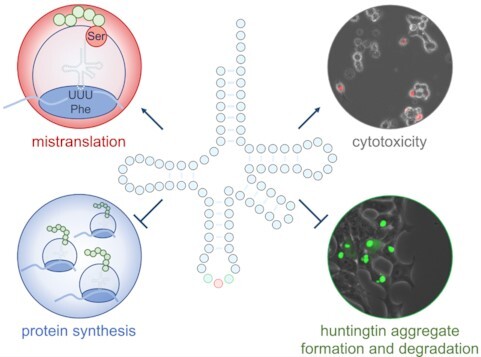
A human tRNA^Ser^ variant found in 1.8% of the population directs serine mis-incorporation and inhibits protein synthesis. Mistranslating cells were drug-resistant and defective in poly-glutamine aggregate clearance in models of Huntington's disease.

## INTRODUCTION

High-fidelity translation of messenger RNAs (mRNAs) was considered essential for life by assuring the functional reproduction of *‘so many highly evolved protein molecules’* (1). In fact, translation of mRNAs is the most erroneous step on the path from gene expression to protein synthesis in cells (2–5). Errors in protein synthesis can result from ribosome stalling or pausing, frameshifting, and amino acid mis-incorporation. Error rates in cells are normally considered low, with 1 mis-incorporation event occurring for every 1000–10 000 codons translated (6,7). Cells can tolerate or even derive a selective advantage from elevated mistranslation rates as a result of stress (8–11), chemical treatment (12–14) or mutations in the protein synthesis machinery (15–18).

The conserved sequence and structure of transfer RNAs (tRNAs) are a major determinant of proteome fidelity. Consequently, single nucleotide substitutions in individual tRNA genes can lead to proteome-wide mistranslation in bacteria (19,20), yeast (18), and mammalian cells (17,21,22). The relevance of tRNA mutations to human disease is becoming more evident since the discovery that cytoplasmic tRNA variants, including those likely to cause errors in translation, are more common in the human population than previously recognized (23). Sequencing efforts, such as the 1000 Genomes Project and our own work (24), confirm that mistranslating tRNA mutants occur in individuals as both rare and common variants with some found in 1–5% of the population.

Mutations in protein coding genes that cause mistranslation, such as aminoacyl-tRNA synthetases, are associated with neurodegeneration in mice (25) and *Drosophila* (26). Mutant tRNAs that lead to a loss of tRNA function were linked to neurodegenerative disease and neuronal phenotypic defects in mice (27,28) and a genetic disorder in humans (29). Mitochondrial tRNA variants have long been associated with human diseases, including neurodegeneration (30). MELAS (mitochondrial myopathy, encephalopathy, lactic acidosis, and stroke‐like episodes) (31) and MERRF (myoclonus epilepsy associated with ragged red fibers) (32) are two major neurodegenerative diseases caused by mutations in mitochondrial tRNAs. In both cases, the mutant tRNAs, which occur at different locations in the tRNA body, cannot be properly post-transcriptionally modified at position 34 of the anticodon. Single mitochondrial tRNA mutants lacking the base 34 taurine modification are less efficient in decoding their cognate codons, resulting in reduced protein synthesis in the mitochondria. The tRNA^Leu^ variants lacking U34 modification can read UUA but not UUG Leu codons (33).

Cytoplasmic tRNAs that specifically cause amino acid mis-incorporation, however, have not been assessed for their contribution to neurodegenerative disease. Recently, loss-of-function cytoplasmic tRNA variants emerged with connections to neurodegeneration (23). The n-Tr20 mutant is a tRNA^Arg^_CTC_ with a C50T substitution in the T-arm of the tRNA (27). The mutation leads to a processing defect and the mature tRNA is not produced. Although there are five nearly identical tRNA^Arg^_CTC_ genes in the genome, the affected tRNA gene normally shows high expression in the cerebellum where the n-Tr20 gene product accounts for 60% of the tRNA^Arg^_CUC_ pool (27). In a screen for neurodegeneration in mice, this tRNA mutant was found to be causative when co-incident with a mutation in the ribosome recycling factor GTPBP2. Together both mutants act to stall the ribosome and reduce the rate of protein synthesis. Mice with these mutations displayed multiple neurodegenerative phenotypes, locomotor defects, and died at 8–9 weeks (27). Further studies of this tRNA^Arg^ mutant found altered synaptic transmission and increased susceptibility to seizures in mice (28).

Mistranslation is also associated with proteotoxic stress and neurodegeneration in mice (25) and patient-derived cell lines (34). Many studies have focused on tRNA synthetase mutants, particularly those defective in editing mis-charged amino acids. One example involves an editing defective AlaRS that mis-charges tRNA^Ala^ with Ser, causing increased levels of misfolded proteins in neurons. Mice expressing the mutant AlaRS displayed reduced body weight and a neurodegenerative phenotype resulting from cell loss and ataxia of Purkinje cells in the cerebellum (25). Another AlaRS editing-defective mutation caused cardioproteinopathy in mice, characterized by protein aggregation in cardiomyocytes, cardiac fibrosis and dysfunction (35). AlaRS mutants are also associated with additional diseases of the nervous system, including Charcot-Marie-Tooth (CMT) disease (36) and early-onset epileptic encephalopathy (37).

Inspired by these studies, we hypothesized that a tRNA variant that also caused mistranslation will act to modify the progression of neurodegenerative disease at the molecular level by affecting protein homeostasis and protein aggregation. We investigated the disease-modifying potential of mistranslating tRNA variants in combination with multiple mammalian cellular models of Huntington's disease (HD). HD, like many other neurodegenerative diseases (38), is characterized by the misfolding and aggregation of specific proteins. Protein misfolding typically has the greatest impact on post-mitotic cells such as those found in the heart, brain, and eye (25,39). Since these cells cannot readily divide or undergo apoptosis, misfolded proteins and protein aggregates accumulate over time, leading to dysregulation of the proteome, cytotoxicity, and eventually cell death (39).

Disorders characterized by protein folding stress or by impaired protein quality control may be particularly susceptible to the effects of mistranslating tRNAs, since mistranslation increases the synthesis of misfolded proteins to further burden the cellular protein folding stress responses (17,21,40). In the case of HD, proteinopathy is triggered by an expanded CAG (Gln) codon repeat in exon1 of the HTT gene encoding the huntingtin protein. Pathogenicity results primarily from a region corresponding to the first exon of the HTT gene (mHTTexon1), which can generate a polyQ expanded Htt protein either by mRNA splicing or proteolysis (41,42). CAG repeats of >38 glutamine residues are associated with disease risk (43). CAG repeat length thereafter correlates with age of onset and severity, but the relationship is highly variable (44). Indeed, some patients with the same CAG repeat length differ by over 20 years in age of onset (44). Further, the severity of symptoms can differ greatly between individuals with a similar age of onset (45). The discrepancies imply the existence of genetic modifiers of Huntington's disease, and several protein-coding genes have been proposed (44). Searches for genetic modifiers, thus far, have relied on whole exome sequencing and single nucleotide polymorphism (SNP) arrays which do not capture tRNA or other non-protein coding RNA gene variants. In addition, whole genome sequencing approaches lack the depth of coverage, read length, and mapping strategy required to confidently identify all tRNA variants in a human genome (24). In this study, we demonstrate that a naturally occurring tRNA variant has significant potential to act as a genetic modifier to Huntington's disease and conceivably other forms of neurodegenerative disease.

## MATERIALS AND METHODS

### Plasmids and strains

Expression constructs, cloning procedures and primers (Supplementary Table S1) are described in supplemental methods. Plasmid DNA for transfection in mammalian cells was purified by Midi-Prep (GeneAid, New Taipei City, Taiwan) from 100 ml *Escherichia coli* DH5α cultures according to manufacturers’ instructions. For all tRNA genes used in our study, we collected data on the folding predictions of the wild-type and mutant tRNAs (gtRNAdb (46)), and available expression data from human cells (gtRNAdb (46), UCSC genome browser (47)). All tRNAs used were predicted to be expressed and fold into a canonical tRNA structure in human cells (Table 1).

**Table 1.** tRNA genes and variants

tRNA gene	Variants	Mistranslation	Variant description	Documented mistranslation	tRNA score^f^ wt;variant		Expression ARM^g^;CHIP^h^	
Ser-AGA-2–3	G35A	Ser at Phe (UUU/C), Leu (UUA) codons	natural MAF = 1.8%^a^	N2A cells^b^	89.6	89.6	+	+
Ser-CGA-2–1	C34T, A36G	Ser at Pro codons	synthetic	yeast^c^ N2A cells^b^	94.0	94.1	+	+
Pro-TGG-1–1	C3G, G70T	Ala at Pro codons	synthetic	yeast^d^ HEK293^e^ cells	74.9	70.8	+	+

^a^Data from 1000 Genomes Project (46,82); ^b^this study; ^c^homolog of Ser-CGA C34T, A36G mistranslation documented in yeast (56,83), ^d^homolog of Pro-TGG-1–1 3G, 70T mistranslation documented in yeast (18), ^e^Pro-TGG-1–1 C3G, G70T mistranslation documented in HEK293 cells (22), ^f^tRNA gene score calculated using tRNA-Scan SE (Infernal score) (46); ^g^ARM = ARM-seq data suggesting expression (46,84); ^h^CHIP = CHIP-seq hits for at least three proteins found in RNA Polymerase III holoenzyme or initiation complex (RPC155, POLR3G, BRF1, BDP1, GTF3C2, TBP) (47,85–88).

### Cell culture and transfection

Experiments were performed in murine Neuro2a Neuroblastoma (N2a; ATCC #CCL-131), human SH-SY5Y neuroblastoma (SH-SY5Y; ATCC #CRL-226), or rat pheochromocytoma (PC12; parent cells: ATCC #CRL-1721)-derived cells. PC12-derived cell lines containing HTT-exon1 fused to EGFP with 23Q or 74Q polyQ under doxycycline promoter (48) were a gift from David Rubinsztein (University of Cambridge, UK). All cell lines were grown at 37°C with humidity and 5% CO_2_. N2a and SHSY5Y cells were cultured in high glucose Dulbecco's modified Eagle medium (DMEM, 4.5 g/l glucose; Gibco by Life Technologies, Carlsbad, CA) containing penicillin (100 IU/ml), streptomycin (100 μg/ml; P/S; Wisent Bioproducts, Montreal, QC, Canada) and 10% fetal bovine serum (FBS; Gibco). PC12-derived cell lines were cultured in high glucose DMEM containing P/S, 10% horse serum (Gibco), 5% FBS (Gibco), 50 μg/ml G418 (Gibco), and 150 μg/ml hygromycin B (Invitrogen, Carlsbad, CA, USA). All transfections were performed using Lipofectamine 2000 transfection reagent (Invitrogen) with 2 μg/ml plasmid DNA, following the manufacturer's instructions.

### Small molecules and peptides

Carbobenzoxy-l-leucyl-l-leucyl-l-leucinal (MG132; Sigma-Aldrich 474790, Darmstadt, Germany) and integrated stress response inhibitor (ISRIB; Sigma-Aldrich SML0843) were dissolved in DMSO and cells were treated with final concentrations as described.

### Cellular viability and toxicity assays

Cellular viability was assessed using a CelltitreGlo 2.0 Luminescent Viability Assay (Promega, Madison, WI) in at least three biological replicates following the manufacturer's instructions. Cytotoxicity was assayed with a CytotoxGlo luminescent cytotoxicity assay (Promega) in at least four biological replicates, following the manufacturer's instructions. Cells were assayed 48 h post-transfection. For assays containing the proteasome inhibitor, cells were treated with MG132 (0.1–10 μM as indicated) or vehicle (DMSO) for 4 h immediately before assay.

### Western blotting

Detailed western blotting procedures are described in supplemental methods. The following primary antibodies were used in this work: α-GFP, Abcam, Cambridge, UK, ab32146; α-GAPDH, Sigma-Aldrich, Darmstadt, Germany, MAB374m; α-HSP70, Invitrogen, MA3-006; α-HSP90, Protein Tech, Rosemont, IL, USA, 13171–1-AP; α-Phospho-eIF2α Ser52, ThermoFisher Scientific, 44–728G; α-eIF2α, ThermoFisher Scientific, AHO0802; α-eEF2, Cell Signaling Technology, Danvers, MA, USA, 2332; α-Phospho-eEF2 (Thr56), Cell Signaling Technology, 2331.

### Mass spectrometry

Detailed mass spectrometry protocols are described in the supplemental methods. Briefly, mCherry protein and wild-type or mistranslating tRNA^Ser^ were co-expressed in N2a cells and mCherry protein was purified using RFP-trap agarose bead immunoprecipitation (Chromotek, Munich, Germany). Immunoprecipitated mCherry was visualized on SDS-PAGE and bands at the correct molecular weight were excised from the gel for MS/MS following tryptic digest of the protein samples. Hits representing Ser misincorporation at Phe codons were curated to include only peptides with a peptide score (–10log *P*) of >50.

### Fluorescence microscopy

Detailed fluorescence microscopy methods are described in supplemental methods. Briefly, Fluorescent microscopy images were captured on an EVOS FL auto fluorescent microscope (Thermo Fisher Scientific). GFP (470 ± 22 nm excitation, 510 ± 42 nm emission) and RFP (531 ± 40 nm excitation, 593 ± 40 nm emission) filter cubes were used to capture green or red fluorescence. An EVOS onstage incubator was used for live cell experiments and images were quantitated using ImageJ/Fiji (49,50) (see supplemental methods, supplemental appendix).

### Cycloheximide chase protein degradation assays

N2a cells were transfected for 48 hr before the experiment in 96-well plates with three biological and six technical replicates. Cells were washed once with Hank's buffered salt solution (HBSS; Gibco by Life Technologies), then media was replaced with DMEM (10% FBS, P/S) containing 50 μg/ml cycloheximide (Sigma-Aldrich) and either 10 μM MG132 or equivalent concentration of DMSO. The plate was immediately transferred to the EVOS FL environment chamber pre-heated to 37°C with 5% CO_2_ and humidity. After acclimatizing the plate for 1 h, images were captured every 30 min for 12 h. Fluorescence was quantitated using a semi-automated approach in ImageJ (see supplemental information). Initially, increasing concentrations of cycloheximide (0, 50, 250 and 500 μg/ml) were also assessed with single fluorescent images with the same approach after 24 h incubation (see supplemental information). All concentrations resulted in a similar reduction in fluorescence after 24 h, so the lowest concentration (50 μg/ml) was selected for the kinetic assay.

### Semi-denaturing detergent agarose gel electrophoresis (SDD-AGE)

N2a cells were transfected as above. In one experiment (Supplementary Figure S6A), cells were incubated with DNA and lipofectamine 2000 for 24 h. In another experiment (Figure 5C–E), cells were incubated for 48 h with lipofectamine 2000 and DNA, then cells were treated for 4 h with either 10 μM MG132 dissolved in DMSO or an equal volume of DMSO. Cell lysates were prepared as above (see Western blotting) and protein concentrations were measured with the BCA assay following manufacturer's instructions. SDD-AGE assays were performed similarly to published approaches (51). Lysate volumes containing 40 μg protein were diluted in 3× loading dye (0.5 M Tris–HCl, pH 6.8; 1.12 M sucrose; 0.025% bromophenol blue; 3.8% SDS) with sterile milliQ H_2_O, loaded and separated on 1.5% agarose gels containing 0.1% SDS. Proteins were transferred to a nitrocellulose membrane by capillary electrophoresis overnight using Tris-acetate-EDTA buffer (40 mM Tris-base, 20 mM acetic acid, 1 mM EDTA) with 0.1% SDS. EGFP-tagged polyQ aggregates were visualized by western blotting with α-GFP antibody (see supplemental information).

### Protein aggregation clearance assay

PC12-derived cells were transfected using lipofectamine 2000 and transfectants expressing wild-type or mutant tRNAs were identified by red fluorescence from a plasmid-encoded mCherry protein. Forty-eight hours post-transfection, mHTT-exon1 74Q-EGFP expression was induced with 2 μg/ml doxycycline (doxycycline hydrochloride; Sigma-Aldrich) as before (48). After 96 h, cells were washed once with doxycycline-free medium and thereafter maintained in doxycycline-free medium. Images were captured daily in RFP (531 ± 40 nm excitation, 593 ± 40 nm emission) and GFP (470 ± 22 nm excitation, 510 ± 42 nm emission) settings from the time at induction to 72 h after doxycycline was removed. To determine the fraction of transfected cells containing aggregates at each time point, the number of transfected cells (red) containing visible aggregates (green) in each image was counted manually by overlaying fluorescent images in ImageJ.

## RESULTS

### Transfer RNA variants used in this study

Transfer RNA variants can elicit several different types of errors in protein synthesis (23). Here, we focused on tRNA variants that specifically cause amino acid misincorporation. The cognate aminoacyl-tRNA synthetases (AARS) for Ser, Ala, and to a lesser extent for Leu, do not use the anticodon as an identity or recognition element (52). Thus, nonsynonymous anticodon variants of tRNA^Ser^, tRNA^Ala^ and tRNA^Leu^ have potential to cause amino acid misincorporation. Seryl-tRNA synthetase is especially flexible in recognizing tRNA^Ser^ with different anticodons (53), since serine is encoded by six codons with no single nucleotide common to all possible sequences. Indeed, engineered tRNA^Ser^ anticodon variants cause mistranslation in mouse and human cells (21), but naturally occurring variants have not been tested.

**Figure 1.**
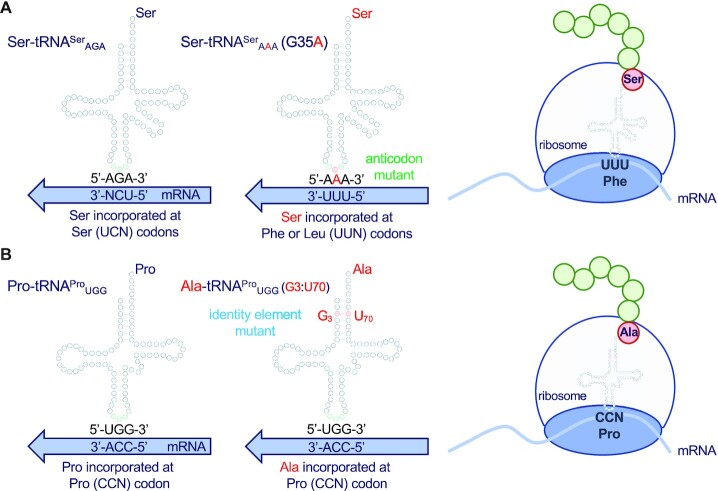
Mechanisms of tRNA-dependent mistranslation. Anticodon (**A**) or identity element (**B**) mutations in tRNAs can lead to mistranslation. Anticodon mutations in tRNA^Ser^ genes (A) lead to a mutant tRNA that still accepts serine and now decodes other codons. The tRNA^Ser^ G35A mutant decodes phenylalanine or leucine (UUN) codons with serine. We also characterized a tRNA^Ser^_UGG_ variant (not shown) that decodes proline codons with serine. Mutations in human tRNAs can lead to the acquisition of a G3:U70 base pair, which is a critical identity element for AlaRS (B). The resulting tRNA^Pro^ (G3:U70) is an efficient alanine but not proline acceptor that retains the ability to decode Pro codons.

By searching the genomic tRNA database (46), we found an uncharacterized tRNA^Ser^ anticodon mutant (tRNA-Ser-AGA-2–3 G35A) that occurs in 1.8% of the sequenced population (Figure 1A) (23). The mutant occurs primarily in the tRNA^Ser^-AGA-2-3 gene and is also found more rarely in the identical tRNA^Ser^-AGA-2-2 gene (23). In an independent targeted sequencing effort covering all human tRNA genes, we identified the same mutant in the tRNA^Ser^-AGA-2-3 gene at a minor allele frequency of 3% in a population of 84 individuals (24). In eukaryotes, tRNA^Ser^_AGA_ is a substrate for A_34_-to-I_34_ editing (54), yielding an IGA anticodon which can decode UCU, UCC and to a lesser extent UCA codons (55). Hence, assuming canonical A:U pairing in the second anticodon:codon position, the tRNA-AGA-2-3 G35A variant has potential to decode UUU (Phe), UUC (Phe), and UUA (Leu) codons. We also investigated two additional mistranslating tRNA variants that we previously characterized in yeast (18,56) and mammalian cells (22) for their ability to direct amino acid mis-incorporation. One variant is caused by mutation of an identity element such that a different AARS recognizes the tRNA (Figure 1B). The resulting Ala-tRNA^Pro^ decodes Pro (CCA/G/U) codons with Ala (18,22). The second variant (tRNA^Ser^ CGA-2-1 C34T, A36G) is a human homolog of a tRNA^Ser^ with a UGG (proline) anticodon that led to mis-incorporation of Ser at Pro codons (CCA/G/U) in yeast (56). Properties of the tRNA genes and variants are noted below (Table 1), while additional gene and SNP identifiers as well as sequences for each tRNA gene locus are listed in the supplemental information (Supplementary Table S2).

### tRNA^Ser^_AAA_-dependent amino acid misincorporation

To confirm that the tRNA^Ser^_AAA_ variant causes the expected Ser incorporation at Phe codons in cells, we immunoprecipitated mCherry protein from cells expressing tRNA^Ser^_AGA_ or tRNA^Ser^_AAA_ and identified multiple peptides corresponding to Ser misincorporation by mass spectrometry. The mCherry coding sequence contains ten UUC codons that may be mistranslated by tRNA^Ser^_AAA_. We detected Ser misincorporation at multiple Phe codons in mCherry from cells expressing tRNA^Ser^_AAA._ In addition, we observed potential mistranslated peptide hits, i.e. probability of random hit score (–10log *P*) ∼40–60, in both normal (Supplementary Figure S1A) and mistranslating cells (Supplementary Figure S1C, Tables 2 and 3). While these lower scoring hits match the full peptide mass, following fragmentation, both hits contain significantly more unidentified peaks and lack multiple y and b ions that cover the site of interest. This is in contrast to the higher scoring hits documenting mistranslation in cells expressing tRNA^Ser^_AAA_, where the spectra have multiple ions with a much higher signal to noise ratio confirming misincorporation (Supplementary Figure S1B, D, E, Table 2). For an overview of misincorporation in the mCherry reporter, we summarized all peptide hits with –10log *P* > 50 for Phe or Ser incorporation at each Phe codon in mCherry (Table 3). The spectral counts indicate a greater level of mis-incorporation of Ser at Phe codons in mistranslating cells (26 Ser / 266 Phe) compared to the background level (6 Ser / 304 Phe) observed in normal cells. Most of the hits in wild-type cells occur at Phe70 (Supplementary Figure S1A), yet these spectra lack sufficient y and b ion support to confirm these peptides as evidence that mis-incorporation occurs in normal cells.

**Table 2.** Selected observed peptides showing Ser mis-incorporation at Phe codons in mCherry

Supplementary Figure S1 panel	tRNA^Ser^ anticodon	Phe codon mCherry	AA change mCherry	Peptide sequence	-10log*P*	Area
A	AGA	UUC	F70S	GGPLPFAWDILSPQ(+.98)SMYGSKA	58.75	1.7 × 10^9^
B	AAA	UUC	F70S	GGPLPFAWDILSPQSM(+15.99)YGSK	94.78	3.8 × 10^7^
C	AAA	UUC	F70S	GGPLPFAWDILSPQ(+.98)SMYGSKA	48.50	2.3 × 10^7^
D	AAA	UUC	F104S	VM(+15.99)NSEDGGVVTVTQDSSLQDGEFIYK	88.19	2.0 × 10^7^
E	AAA	UUC	F123S	VM(+15.99)NFEDGGVVTVTQDSSLQDGESIYK	92.40	2.0 × 10^7^

S indicates Ser misincorporation at Phe codons. Spectra for these peptides are shown in Supplementary Figure S1.

**Table 3.** Observed spectral counts for Ser or Phe incorporation at Phe codons in mCherry

	tRNA^Ser^_AGA_		tRNA^Ser^_AAA_	
Phe codon position in mCherry	No. Phe peptides	No. Ser peptides	No. Phe peptides	No. Ser peptides
32	122	1	86	5
61	18	0	15	1
70	14	4	9	9
92	5	0	5	2
96	0	0	0	0
104	54	1	36	4
123	55	0	36	5
134	36	0	42	0
**Total:**	**304**	**6**	**229**	**26**

Peptides hits with –10log *P* score >50 are shown.

### Reduced protein levels in mistranslating cells

**Figure 2.**
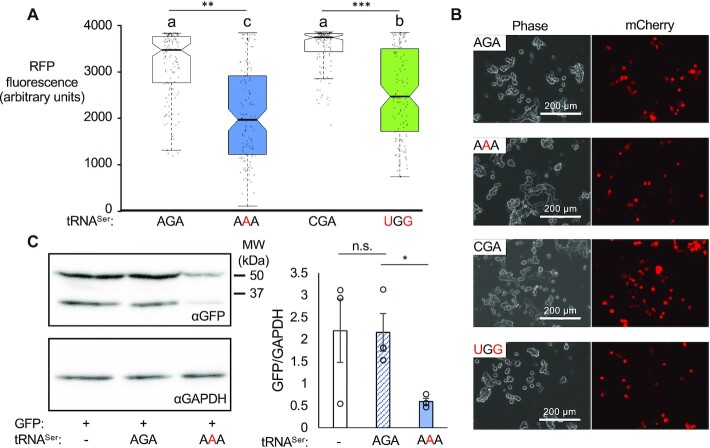
Fluorescence and expression of mCherry protein in mistranslating cells. N2a cells were transfected with a plasmid encoding human tRNA^Ser^_AGA_ or G35A variant tRNA^Ser^_AAA_; or tRNA^Ser^_CGA_ or the C34G, A36G variant tRNA^Ser^_UGG_ and fluorescently dead GFP (S65F or P) fused to an active mCherry transfection marker. Fluorescence of cells was measured by fluorescence microscopy (RFP, ex 531 nm, em 593 nm). Each box represents data from 135 cells from three biological and nine technical replicates (**A**). Midline represents the median, boxes represent quartiles, and whiskers represent 1.5× the interquartile range. Representative images were captured under 20× magnification with phase or RFP settings (**B**). Cell lysates were harvested and western blotted for fluorescent protein expression with anti-GFP and anti-GAPDH antibodies as a loading control (**C**). Anti-GFP blots were quantitated in three biological replicates by densitometry normalized to GAPDH. Stars indicate *P*-values from independent sample t-tests (n.s. = no significant difference, * *P* < 0.05, ** *P* < 0.01, *** *P* < 0.001) and letters indicate significantly different groups determined by Tukey's Honestly Significant Different (HSD) test, where groups sharing a letter are not significantly different and groups not sharing a letter are significantly different (α = 0.05).

Previous reports established a translation-inhibition response to mistranslating tRNAs expressed in mammalian cells (21,40). Through inhibition of mRNA translation initiation or elongation, general protein synthesis can be downregulated in response to mistranslation or tRNA dysfunction (21,57). Using fluorescence microscopy, we measured red fluorescence (ex. 542 ± 20 nm, em. 593 ± 40 nm) of N2a cells expressing tRNA^Ser^_AGA_, tRNA^Ser^_AAA_, tRNA^Ser^_CGA_ or tRNA^Ser^_UGG_ and mCherry. We observed a significant reduction in fluorescence of cells expressing either tRNA^Ser^ mutant compared to wild-type tRNA (Figure 2A, B). The transfections were repeated with plasmids expressing wild-type tRNA^Ser^_AGA_ or the tRNA^Ser^_AAA_ variant for western blotting analysis of GFP (S65F)-mCherry expression (see supplementary methods). Compared to a GAPDH control, the GFP-mCherry protein levels were reduced > 3.6-fold in mistranslating cells (Figure 2C). We captured images of HEK239 cells expressing wild-type and mistranslating tRNA^Pro^ G3:U70 with an EGFP reporter as before (22). Analysis of these images confirmed that tRNA^Pro^ G3:U70 does not elicit the translation suppression response (Supplementary Figure S2A) that we observed with the anticodon variants of tRNA^Ser^.

### tRNA-dependent toxicity in human and mouse cellular models of HD

**Figure 3.**
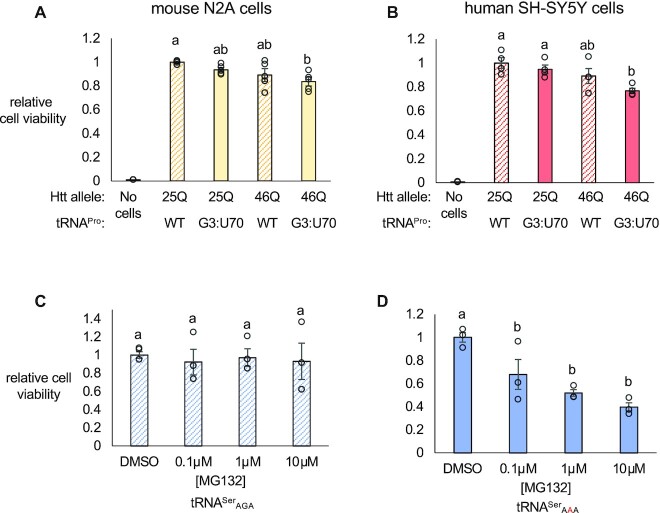
Toxic interactions of tRNA variants with a deleterious polyQ allele or proteosome inhibition. N2a (**A**) or SHSY5Y cells (**B**) were co-transfected with two plasmids encoding human tRNA^Pro^_UGG_ or the G3:U70 variant and HTT-exon1 containing 25 (25Q) or 46 (46Q) CAG/CAA mixed codon repeats encoding polyQ. Cellular viability was measured 24 h post-transfection with CellTitreGlo 2.0. Luminescence readings were normalized to the ‘25Q WT’ control. N2a cells were transfected with a plasmid encoding human tRNA^Ser^_AGA_ (**C**) or G35A variant tRNA^Ser^_AAA_ (**D**) and HTT-exon1 containing 23Q fused to EGFP as a transfection marker. Cellular viability was assayed 48 h post-transfection with the CellTitreGlo 2.0 assay following 4 hr treatment with increasing concentrations of MG132 or equal concentration of DMSO. Luminescence readings were normalized to the DMSO controls. Letters indicate significantly different groups determined by Tukey's HSD test (α = 0.05). Error bars represent the mean ± 1 standard deviation of at least three biological replicates.

To investigate the viability of cells expressing a known mistranslating tRNA combined with an aggregating polyQ allele, we co-transfected plasmids encoding a wild-type or G3:U70 human tRNA^Pro^ with a non-pathogenic (25Q) or mildly pathogenic (46Q) version of HTT-exon1 (58). The experiments were performed in murine N2a and human SHSY5Y cells. Both are neuroblastoma-derived lines that are routinely used as a model for protein misfolding disease, including HD (48,59). Using a luminescent assay for cell viability (Celltitre Glo 2.0), we observed no significant loss of viability from the mutant tRNA alone or from the mildly deleterious HTT-allele co-expressed with a wild-type tRNA. Only the combination of HTT-exon1 46Q expression in mistranslating cells resulted in a significant reduction (1.3 ± 0.05-fold in SHSY5Y; 1.2 ± 0.06-fold in N2a) in cellular viability compared to cells expressing wild-type tRNA and 25Q (Figure 3A, B). The data demonstrate a synthetic toxic interaction between the Ala accepting tRNA^Pro^ G3:U70 mutant and a deleterious HTT allele. We observed the same result in both mouse (Figure 3A) and human cells (Figure 3B).

### Interactions of polyQ HTT alleles with tRNA^Ser^ mutants

We cloned the tRNA-Ser-AGA-2–3 wild type and G35A variant and tRNA-Ser-CGA-2–1 wild type and C34G, A36G double mutant with native genomic context (±300 bp) into plasmids containing mCherry fused to a fluorescence-inactivated GFP variant (see supplemental methods). In N2a cells, we co-transfected plasmids with tRNA^Ser^_AAA_, tRNA^Ser^_UGG_ or the respective WT tRNA controls along with the 23Q or 46Q HTT-exon1 allele. Unlike our studies with tRNA^Pro^ G3:U70, the tRNA^Ser^ anticodon variants showed no significant loss of cellular viability on their own or in combination with the 46Q HTT-exon1 (Supplementary Figure S3A, B).

For the following experiments, we focused our investigations on tRNA^Ser^_AGA_ and the G35A variant, since the variant occurs naturally in the human population and leads to phenotypic defects, including inhibited protein synthesis. We cloned the tRNA^Ser^_AGA_ and the tRNA^Ser^_AAA_ variant genes into plasmids expressing 23Q or 74Q HTT-exon1 allele fused to EGFP. We used a CytotoxGlo assay to measure cellular toxicity. In this assay, the tRNA^Ser^_AAA_ variant was significantly cytotoxic compared to the wild-type tRNA^Ser^_AGA_, but the mutant tRNA showed no apparent additional toxic effect in combination with 74Q HTT-exon1 (Supplementary Figure S3C, D).

We hypothesized that inhibition of the proteasome would increase the toxicity of mistranslating cells because of the accumulation of mistranslated and misfolded proteins. We used increasing concentrations of MG132 in cells expressing 23Q HTT-exon1-EGFP and either tRNA^Ser^_AGA_ (Figure 3C) or the tRNA^Ser^_AAA_ variant (Figure 3D) and measured cell viability. We observed a MG132 concentration-dependent decrease in cell viability only in cells expressing the mistranslating tRNA variant, demonstrating a synthetic toxic interaction between the naturally occurring tRNA^Ser^_AAA_ mutant and proteasome inhibition. MG132 treatments at the same concentrations had no effect on the viability of cells expressing wild-type tRNA (Figure 3C).

Anticipating that proteasome inhibition would exacerbate toxicity of both the mistranslating tRNA and the 74Q allele, we measured the toxicity of cells with or without treatment of MG132. We again confirmed cytotoxicity resulting from the tRNA^Ser^_AAA_ variant in comparison to wild-type tRNA, however, we observed no additional toxicity with 74Q compared to the 23Q HTT-allele in mistranslating cells (Supplementary Figure S3D). Compared to normal conditions (Supplementary Figure S3C), we note that the addition of MG132 (Supplementary Figure S3D) resulted in a greater and more significant increase in cytotoxicity for mistranslating cells compare with wild type cells.

### Kinetics of polyQ aggregate formation in mistranslating cells

**Figure 4.**
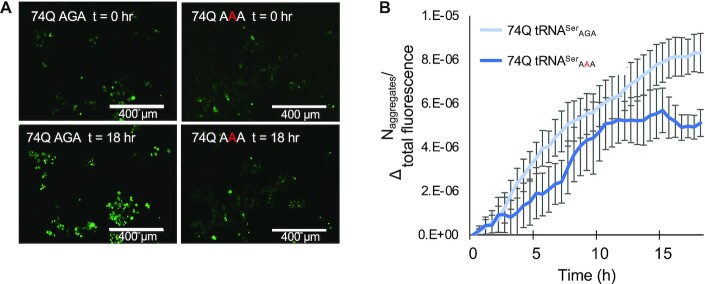
Formation of polyQ aggregates in mistranslating cells. N2a cells were transfected with a plasmid encoding human tRNA^Ser^_AGA_ or G35A variant tRNA^Ser^_AAA_ and HTT-exon1 containing 74Q-EGFP. Images were captured beginning 24 h post-transfection by fluorescence microscopy in GFP settings at 10× magnification every 30 min for an 18 h time course. (**A**) Representative images from the beginning (*t* = 0 h) and end (*t* = 18 h) of the time course are shown. (**B**) The number of aggregates in each image was quantitated in ImageJ (see supplemental information). The number of aggregates in each image of the series was then normalized to total fluorescence of the same image (*N*_aggregates_/total fluorescence), and initial values were subtracted (Δ*N*_aggregates_/total fluorescence). Error bars represent the mean ± 1 standard deviation of six biological replicates.

Using live cell fluorescence microscopy, we captured the formation of EGFP-tagged polyQ aggregates in N2a cells expressing either tRNA^Ser^_AGA_ or the tRNA^Ser^_AAA_ variant over an 18 h time-course. We quantified the fluorescence and number of aggregates in each image series using a semi-automated approach in ImageJ (Supplementary Figure S4). Mistranslating cells accumulated fewer 74Q aggregates and less overall fluorescence signal over the time-course (Figure 4A, Supplementary Figure S5, Data File S1). We normalized the number of aggregates in each image to total fluorescence of the image to account for the reduced fluorescence in mistranslating cells and variability in the number of fluorescing cells between images. In cells expressing the wild-type tRNA, the number of aggregates per unit fluorescence increased over time. In mistranslating cells, the appearance of aggregates proceeded at a slower rate and plateaued earlier at the 10-h time point (Figure 4B). To further validate that the observed reduction in fluorescence was due to expression of the mutant tRNA, we co-transfected plasmids expressing mCherry and the 23Q HTT-exon1 allele fused to EGFP, with the mutant tRNA^Ser^_AAA_ encoded on either one plasmid or the other. Regardless of which plasmid the tRNA was expressed from, 23Q-EGFP fluorescence was significantly and equivalently reduced compared to cells expressing no additional tRNA, and the effect was maintained for at least 48 hrs post-transfection (Supplementary Figure S2B).

### Mistranslating cells accumulate smaller and fewer polyQ aggregates

**Figure 5.**
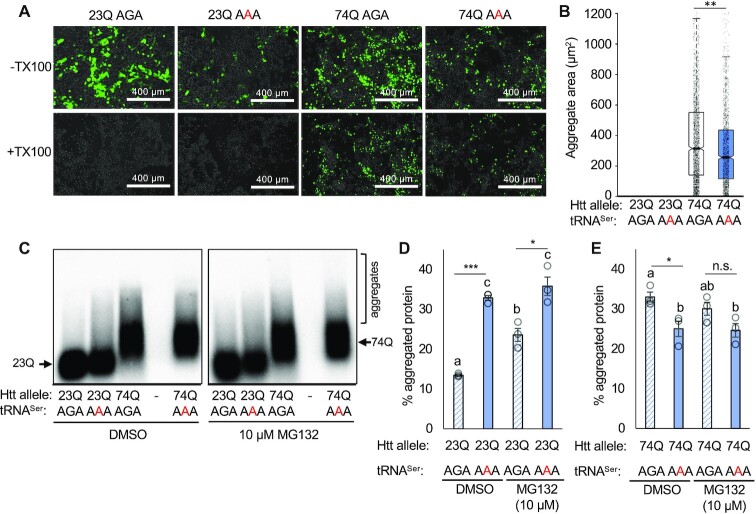
Insoluble PolyQ aggregate size and levels in mistranslating cells. N2a cells were transfected with a plasmid encoding human tRNA^Ser^_AGA_ or G35A variant tRNA^Ser^_AAA_ and HTT-exon1 containing 23Q or 74Q fused to EGFP. (**A**) Representative images were captured by fluorescence microscopy before and after Triton X-100 treatment at 10× magnification. Images are overlaid from GFP and phase settings. (**B**) The area of fluorescent bodies remaining after detergent was measured in ImageJ (see supplemental methods). Midline represents the median, boxes represent quartiles, and whiskers represent 1.5× the interquartile range. (**C**) Cell lysates were harvested from N2a cells transfected with the same plasmids and analyzed by SDD-AGE and western blotting (α-GFP). Cells were treated for 4 h with 10 μM MG132 or an equivalent volume of DMSO. Higher molecular weight smears in the 74Q lanes indicate the presence of aggregated proteins. The SDD-AGE images were quantified for (**D**) 23Q and (**E**) 74Q aggregates by densitometry (see Supplementary Figure S6). Intensity of the high molecular weight aggregates was normalized to total intensity and expressed as a percentage. Error bars represent mean ± 1 standard deviation from 3 biological replicates. Stars indicate *P*-values from independent sample t-tests (n.s. = no significant difference, * *P* < 0.05, ** *P* < 0.01, *** *P* < 0.001). Letters indicate significantly different groups determined by Tukey's HSD test, where groups sharing a letter are not significantly different and groups not sharing a letter are significantly different (α = 0.05).

To assess the effects of a mistranslating tRNA on insoluble polyQ aggregate formation, we performed a membrane detergent assay to quantify insoluble EGFP-HTT-exon1 polyQ aggregates in cells expressing either tRNA^Ser^_AGA_ or the tRNA^Ser^_AAA_ variant. Aggregates were allowed to from over a 48 h transfection period, after which cells were treated with Triton X-100 to permeate the cell membrane. Large, insoluble fluorescent aggregates are retained in the cell, whereas soluble polyQ or small oligomers defuse into the media (Figure 5A) (60). We used thresholding analysis to assess the number and size of aggregates in images captured before and after Triton X-100 treatment. All fluorescent foci disappeared from cells expressing the non-aggregating 23Q-EGFP following Triton X-100 treatment, while the 74Q foci were clearly visible (Figure 5A). Foci remaining in the mistranslating cells were significantly smaller than cells expressing wild-type tRNA, with a median area of 257 μm^2^ compared to 315 μm^2^ in the wild-type tRNA-expressing cells (Figure 5B).

We further investigated the effect of the tRNA^Ser^_AAA_ variant on aggregate formation using semi-denaturing detergent agarose gel electrophoresis (SDD-AGE) (51). SDD-AGE is a semi-quantitative method to visualize insoluble protein aggregates as high molecular weight products after agarose gel electrophoresis and western blotting. We used an α-GFP antibody to detect the EGFP-tagged polyQ proteins. At 24 h post-transfection, we observed less aggregated 74Q protein in cells expressing the mistranslating tRNA compared to wild type, and no evidence of aggregated 23Q-EGFP protein in cells expressing either tRNA (Supplementary Figure S6).

To further promote formation of protein aggregates, we transfected cells for 48 hrs and then treated them for 4 h with MG132 or DMSO as a control. With 48 h transfections, high molecular weight aggregates were observed even in 23Q-expressing cells (Figure 5C, D). Comparing the wild-type tRNA^Ser^_AGA_ and mistranslating tRNA^Ser^_AAA_, we observed different effects on 23Q and 74Q aggregation behavior. Cells expressing 23Q with the wild-type tRNA showed a small fraction of aggregated polyQ in the SDD-AGE assay with 13% of the HTT-23Q protein aggregated. In 23Q-expressing cells with the mistranslating tRNA, however, we found a significant increase in the proportion of aggregated protein (33%) compared to cells expressing the wild-type tRNA (Figure 5C, D). The data suggest that mistranslation of the HTT-23Q allele contributes to an increase in aggregation of the non-deleterious HTT allele.

Cells expressing either wild type or mutant tRNA both showed aggregation of the 74Q protein, but mistranslating cells displayed a smaller fraction of high molecular weight 74Q aggregates (Figure 5C, E) in agreement with our Triton-X100 treatments (Figure 5A, B). Proteasome inhibition with MG132 had no significant effect on aggregation of either 23Q or 74Q in mistranslating cells. We observed a greater accumulation of 23Q aggregates in MG132-treated cells expressing wild-type tRNA, which was still significantly less than the level of 23Q aggregates seen in mistranslating cells (Figure 5D). MG132-treated cells expressing the wild type tRNA and 74Q showed an intermediate level of protein aggregation, which was not significantly different from either untreated wild-type cells or from cells expressing the mistranslating tRNA (Figure 5E). The absence of any change in protein aggregates in mistranslating cells with or without MG132 treatment suggests that the mistranslating tRNA has a dominant effect on huntingtin protein aggregation that is independent of proteasome activity.

### Heat shock protein levels in mistranslating cells

Heat shock protein production is a common cellular stress response mounted to mitigate the toxic effects of misfolded proteins in cells and is known to be activated by mistranslating tRNAs in yeast (18,56). Increased heat shock protein levels were also observed in mice expressing editing-defective aminoacyl-tRNA synthetases (25), and mammalian cells expressing mistranslating tRNAs after extended transfection periods of up to 72 h (21). In a previous study, we observed no change in HSP70 or HSP90 levels in HEK293 cells expressing tRNA^Pro^ G3:U70 (22). Here, we measured the level of heat shock response factors HSP70 and HSP90 24 h after transfection in cells expressing tRNA^Ser^_AAA_ or wild-type tRNA^Ser^_AAA_. Consistent with our previous study on tRNA^Pro^ G3:U70, we did not observe any evidence of increased HSP levels at 24 h (Supplementary Figure S7A). We also measured HSP70 levels after more extended transfections periods (24, 48 and 72 h) and observed no significant differences between wild-type and mistranslating cells (Supplementary Figure S7B).

### Regulation of translation initiation and elongation in mistranslating cells

Previous studies established that certain tRNA anticodon variants expressed in mammalian cells lead to increased phosphorylation of eIF2α at Ser52 (21,28,40). Phospho-Ser52 in eIF2α prevents translation initiation of most cellular mRNAs and is a converging point of the integrated stress response stimulated by numerous cellular stresses. However, p-eIF2α levels vary substantially depending on the tRNA gene variant expressed (21), and how long cells have been expressing the tRNA variant (40). We used western blotting to measure p-eIF2α, eIF2α and GFP levels from cells expressing all combinations of EGFP-fused HTT-exon1 23Q and 74Q with wild-type tRNA^Ser^_AGA_ or the tRNA^Ser^_AAA_ variant. Despite a reduction in the EGFP-fused huntingtin protein in all mistranslating cells, we did not observe an increase in the level of p-eIF2α in mistranslating cells, even after longer transfection periods (Supplementary Figure S8A).

Cells can also down-regulate mRNA translation at the level of elongation. Eukaryotic elongation factor 2 (eEF2) is involved in ribosome repositioning and movement of tRNA from the A-site to P-site during translation (61). Phosphorylation of the eukaryotic elongation factor 2 (eEF2) by eEF2 kinase (eEF2K) at Thr56 reduces translation elongation rates in conditions of nutrient deprivation or various other forms of cellular stress, including proteasome inhibition with MG132 (62). We used western blotting to measure p-eEF2 and eEF2 levels from cells expressing an mCherry transfection marker and tRNA^Ser^_AGA_ or the tRNA^Ser^_AAA_ variant with or without treatment with 10 μM MG132 (Supplementary Figure S8B). MG132 treatment stimulated a significant increase in p-eEF2 levels in both cell lines (Supplementary Figure S8), but we did not observe any difference in p-eEF2 levels in the mistranslating cells compared to wild type. In normal cells, MG132 inhibits both protein degradation (see Figure 7C) and protein synthesis, leading to no change in the steady state protein levels. Despite the clear induction of p-eEF2 in MG132-treated cells, mistranslating cells show a severely reduced level of protein synthesis in conditions that have high or low p-eEF2 (Supplementary Figure S8C).

### Mistranslating cells are resistant to the integrated stress response inhibitor

To further probe the integrated stress response in cells expressing tRNA^Ser^_AAA_, we tested whether the reduction in protein levels could be reversed with a p-eIF2α antagonist. The integrated stress response inhibitor, ISRIB, relieves translation suppression caused by eIF2α phosphorylation. ISRIB promotes the formation of active heterodecameric eIF2B complexes to stimulate eIF2B-dependent translation initiation (63). Cells transfected with plasmids expressing EGFP-fused HTT-exon1 23Q and no tRNA, wild-type tRNA^Ser^_AGA_, or the tRNA^Ser^_AAA_ variant were treated with 500 nM ISRIB over an 18 h time-course. Fluorescent images were captured throughout the time course, and a cytotoxicity assay was completed at the final timepoint.

**Figure 6.**
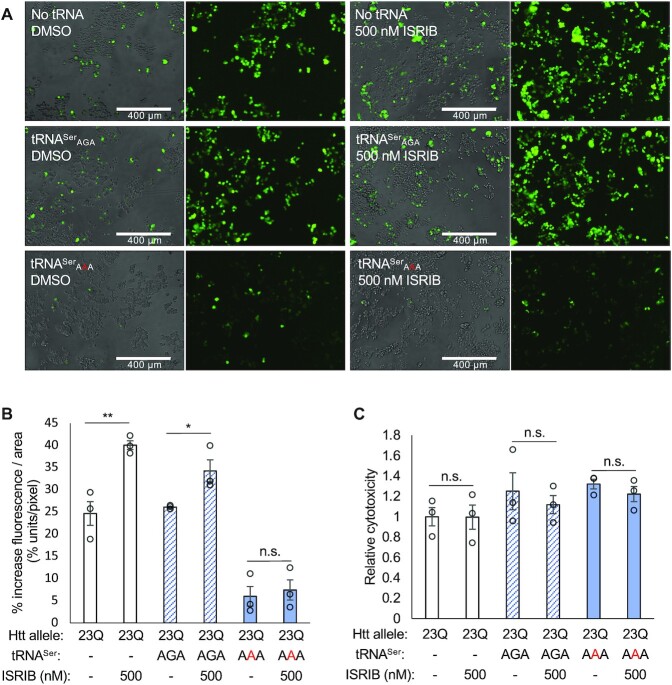
Fluorescence and cytotoxicity of mistranslating cells treated with p-eIF2α antagonist ISRIB. N2a cells were transfected with a plasmid encoding no tRNA, human tRNA^Ser^_AGA_ or G35A variant tRNA^Ser^_AAA_ and HTT-exon1 23Q-EGFP. Cells were treated for 18 h with 500 nM ISRIB or equivalent concentration of DMSO. (**A**) Representative images were capture by fluorescence microscopy in GFP settings after 18 h ISRIB treatment. (**B**) Fluorescence intensity per cell area of six technical and three biological replicates was quantitated in ImageJ (see supplemental methods) before and after treatment. Each bar represents mean (±1 standard deviation) increase in fluorescence intensity per area as a percentage. (**C**) Cytotoxicity after treatment was measured with CytotoxGlo. Luminescence readings were normalized to the ‘23Q −/−’ control. Stars indicate *P*-values from independent sample t-tests (n.s. = no significant difference, * *P* < 0.05, ** *P* < 0.01, *** *P* < 0.001).

Unlike normal cells, mistranslating cells were unable to increase protein synthesis levels in response to ISRIB. In cells expressing no ectopic tRNA, fluorescence of the EGFP-fused HTT-allele increased significantly from the beginning to end of the time course by 25% in DMSO control and 40% in 500 nM ISRIB. In cells expressing wild-type tRNA^Ser^_AGA_, fluorescence increased by 26% in DMSO control and 34% in 500 nM ISRIB. In cells expressing tRNA^Ser^_AAA_, ISRIB did not significantly increase protein levels, with only a 6% increase of 23Q-EGFP production in the DMSO control, and a similar 7% increase in 500 nM ISRIB. Hence, the addition of ISRIB did not simulate synthesis of the 23Q-EGFP huntingtin protein in mistranslating cells (Figure 6A, B). As anticipated, ISRIB also had no effect on the cytotoxicity of the mistranslating tRNA (Figure 6C).

### Defective protein turnover and aggregate clearance in mistranslating cells

**Figure 7.**
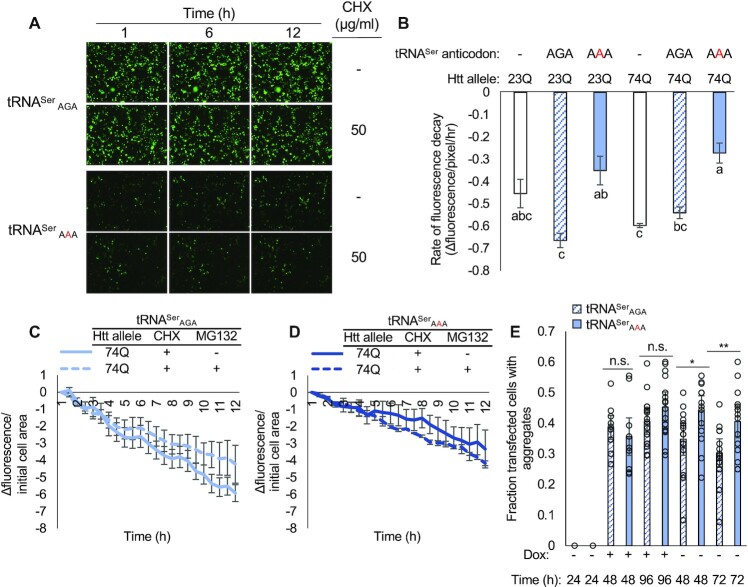
Protein turnover and clearance of PolyQ aggregates in mistranslating cells. N2a cells were transfected with a plasmid encoding human tRNA^Ser^_AGA_ or G35A variant tRNA^Ser^_AAA_ and HTTexon1 containing 23Q or 74Q fused to EGFP for 48 h before cycloheximide chase assays (A−D). Cells were treated with cycloheximide and/or MG132 and fluorescence was captured by live cell fluorescence microscopy. (**A**) Representative images of 23Q-EGFP/tRNA^Ser^_AGA_ and 23Q-EGFP/tRNA^Ser^_AAA_ expressing cells at the indicated timepoints. Fluorescence was quantitated in ImageJ (see supplemental methods). Average rate of protein decay (**B**) is shown as a change in fluorescence normalized to initial cell area (**C**, **D**) (see supplemental information). (**E**) PC12 cells with genomically integrated HTT-exon1 74Q-EGFP under a doxycycline (Dox)-inducible promoter were transfected with a plasmid encoding human tRNA^Ser^_AGA_ or G35A variant tRNA^Ser^_AAA_ and mCherry transfection marker. HTT-exon1 expression was induced with doxycycline 48 h post-transfection and cells were imaged daily by fluorescence microscopy. After 96 h, Dox was removed and daily imaging was resumed for 72 h. Aggregate counting is described in methods (see Supplementary Figure S10). Error bars represent the mean ±1 standard deviation of at least three biological replicates and nine technical replicates each. Stars indicate *P*-values from independent sample t-tests (n.s. = no significant difference, * *P* < 0.05, ** *P* < 0.01, *** *P* < 0.001) and letters indicate significantly different groups determined by Tukey's HSD test (α = 0.05).

Given the synthetic toxic effect we observed in mistranslating cells treated with MG132, and previous reports of tRNA anticodon variants promoting increased proteasome activity (40), we assayed protein turnover in mistranslating cells. Cycloheximide chase assays (64) were performed on N2a cells expressing no tRNA, wild-type tRNA^Ser^_AGA_, or the tRNA^Ser^_AAA_ variant with EGFP-fused HTT-Exon1 23Q and 74Q (Figure 7A-D). Cycloheximide concentrations of 50, 250 and 500 μg/ml were effective in inhibiting translation and promoting turnover of the 23Q-EGFP protein in N2a cells (Supplementary Figure S9). Treatment with MG132 to inhibit the proteasome was used as a negative control as described (64). In cells expressing the tRNA^Ser^_AAA_ variant (Figure 7B, D), we observed a significantly lower rate of protein turnover of both the HTT-exon1 23Q-EGFP and 74Q-EGFP alleles compared to cells expressing wild-type tRNA^Ser^_AGA_ (Figure 7B, C). Compared to cells expressing no plasmid-borne tRNA, turnover of the aggregating 74Q-EGFP but not 23Q-EGFP was significantly reduced in mistranslating cells (Figure 7B). In normal cells, MG132 lead to a reduced rate or protein degradation (Figure 7C), while in mistranslating cells MG132 was not able to further slow their already defective rate of protein degradation (Figure 7D).

To assess whether 74Q aggregate clearance was reduced in an independent polyQ model, we used a rat PC12-derived cell line with a stable, genome-integrated HTT-exon1 74Q fused to EGFP under control of a doxycycline inducible promoter (48). We transfected either tRNA^Ser^_AGA_ or the tRNA^Ser^_AAA_ variant on plasmids with an mCherry transfection marker and monitored the appearance and disappearance of aggregates by induction and removal of doxycycline. Using mCherry to identify tRNA-transfected cells, we counted the number of transfected cells expressing either wild-type or mutant tRNAs containing at least one aggregate (Supplementary Figure S10). The number of cells containing aggregates was not significantly different during doxycycline treatment, but after doxycycline was removed, aggregates persisted significantly longer in cells expressing the mistranslating tRNA (Figure 7E). Indeed, cells with wild-type tRNA had a significantly reduced number of polyQ aggregates at 48 and 72 h after removal of doxycycline, yet we did not observe any decrease in polyQ aggregates in the mistranslating cells following removal of doxycycline. The data suggest that mistranslating cells are defective in clearing protein aggregates that cause disease.

## DISCUSSION

### Huntingtin protein aggregation in mistranslating cells

Our data show that huntingtin aggregates form readily in mistranslating cells, but at a slower rate than in wild-type cells. Since cells expressing the 74Q and mistranslating tRNA strongly suppress protein synthesis, polyQ protein concentrations are less likely to reach the threshold required to seed aggregates (65). Consistent with the translation suppression response in mistranslating cells, we observed fewer and smaller aggregates in cells expressing HTT-exon1 74Q and the tRNA^Ser^_AAA_ variant compared to wild-type tRNA^Ser^. Further, in N2a cells and in an inducible PC12 cell line of HTTexon1 74Q, we observed a greater persistence of aggregates in cells expressing tRNA^Ser^_AAA_ compared to wild-type tRNA^Ser^. We also found that mistranslating cells were generally defective in their ability to degrade proteins. While mistranslating cells synthesize aggregating proteins more slowly, they are also defective in their ability to degrade huntingtin protein aggregates. In these cellular models of neurodegenerative disease, the data indicate that natural mistranslating tRNA variants have the potential to affect the onset, progression, and severity of HD.

The Ser mis-incorporation at Phe codons we observed in cells expressing tRNA^Ser^_AAA_ led to the accumulation of misfolded proteins. The label-free MS/MS approach we used is admittedly not ideal for quantifying the mistranslation rate, which will be the subject of future work. The data suggest an estimate for the rate of Ser mis-incorporation at Phe codons could be ∼9% per UUC codon, which is 90−900 times more than the generally accepted translation error rate of 1 mistake per 1000−10 000 codons (6,7). Although spectral counting may overestimate the error rate, even modest levels of mis-incorporation can lead to the accumulation of mis-made protein as we found. Although mistranslating cells were slower in forming HTT-exon1 74Q aggregates, we observed the normally non-aggregating 23Q protein form a significant amount of protein aggregate in mistranslating cells. While 23Q HTT-exon1 is less aggregation prone than 74Q, *in vitro* studies have demonstrated that 23Q and shorter HTT-exon1 polyQ peptides are capable of aggregation (66). In mistranslating cells, we observed 23Q aggregates at 48 h post-transfection. The data suggest that compared to 74Q, higher expression levels and longer times were needed for the 23Q-EGFP protein to aggregate in mistranslating cells. We also observed increased 23Q aggregation in cells treated with the proteasome inhibition. Our observations on huntingtin protein aggregation suggest mistranslating cells are compromised in their ability to both synthesize and degrade protein aggregates.

### Huntingtin protein aggregation and defects in protein quality control

Our work is the first to explore the interaction between mistranslation and huntingtin polyQ aggregation. Our studies, focused on mistranslation resulting from a tRNA mutant, suggest that other routes to amino acid mis-incorporation, e.g. editing-defective AARS variants, can also exacerbate polyQ aggregation and HD. Interactions between other factors regulating protein homeostasis and polyQ aggregation represent a continuing theme in yeast and mammalian cell models of polyQ aggregation and toxicity. For example, work in yeast shows that molecular chaperones (67,68) and protein degradation (69) are critical systems to combat polyQ aggregation. In Drosophila S2 cells expressing long 138Q, but not shorter polyQ alleles, protein synthesis is downregulated via the translation regulator Orb2 (70). Studies using N2a cells, as we used, examined production and aggregate formation of HTT alleles with 18Q, 64Q or 150Q. Aggregates were monitored over a 48 h period, and in agreement with our study, no toxicity from the 64Q or even the 150Q HTT allele was observed (71). In HeLa cells, the same authors used a reporter for nuclear retention of ribosomal protein S2, which signifies inhibition of ribosome biogenesis. Cells expressing a 97Q allele showed a 3-fold increase in nuclear localization of ribosomal protein S2, demonstrating defective ribosome biogenesis in cells expressing an aggregating HTT allele (71). The authors did not directly measure the rate of protein synthesis, but their data suggest dysregulation of protein synthesis in cells expressing long polyQ alleles. Our data indicate that tRNA variants, which compromise protein homeostasis, may exacerbate the dysregulation of protein synthesis caused by deleterious and longer polyQ alleles.

### Compromised proteostasis in cells expressing mistranslating tRNAs

We found that cells expressing the naturally occurring tRNA^Ser^_AAA_ variant were characterized by several phenotypic defects. Using mass spectrometry, we confirmed amino acid mis-incorporation of Ser at multiple Phe codons. Mistranslating cells were characterized by increased cytotoxicity, general inhibition of protein synthesis, sensitivity to proteosome inhibition, and resistance to the neuroprotective stress response inhibitor ISRIB. Together the data demonstrate that tRNA^Ser^_AAA_ expression leads to a loss of protein homoeostasis.

Despite a mistranslation rate of ∼2–3% Ala incorporation at Pro codons, tRNA^Pro^ G3:U70 is not toxic when expressed alone (22). Here, we found that toxicity of tRNA^Pro^ G3:U70 is evident in combination with a mildly deleterious 46Q HTT-exon1 allele. The data suggest that some mistranslating tRNA variants can exacerbate polyQ toxicity in a model of neurodegenerative disease. Conversely, the naturally occurring tRNA^Ser^_AAA_ variant was toxic alone, but did not show additional toxicity with aggregating huntingtin protein. The differences in toxicity may depend on how cells respond to different types of mistranslation. We observed a consistent reduction in protein synthesis in cells expressing the tRNA^Ser^_AAA_ or tRNA^Ser^_UGG_ variants, but not in cells expressing tRNA^Pro^ G3:U70. Furthermore, cells mis-incorporating Ser at Phe codons showed a stronger repression of translation than cells mistranslating Pro codons with Ser. The severely inhibited protein synthesis observed in cells with tRNA^Ser^_AAA_ may indeed protect the cell from the toxicity of the aggerating huntingtin allele and amino acid mis-incorporation.

A recent study in yeast demonstrated that tRNA variants that result in different types of amino acid change can elicit distinct cellular responses (72). A tRNA^Ser^ mutant that mistranslates Arg codons and a yeast homolog of the same tRNA^Pro^ G3:U70 variant we tested here were found to mistranslate at similar levels. In RNA sequencing experiments, however, each tRNA-expressing strain elicited distinct changes in the transcriptome. For example, down-regulation of genes involved in cell cycle, DNA replication, transcription, and response to stress was observed in cells mistranslating Pro codons with Ala but not in cells mis-incorporating Ser at Arg codons (72).

Phenotypic differences observed from expressing different tRNA variants may also depend on the compatibility of the tRNA in question with a given gain-of-function mutation. For example, nucleotides in and adjacent to the anticodon sequence are often conserved within tRNA isoacceptor groups (46), and can be modified to ensure optimal fidelity and efficiency in recognizing certain codon sets. This is true for wild-type tRNA^Phe^, wherein modified guanine bases in position 34 (73) of the anticodon and the anticodon-adjacent position 37 (74) are used to efficiently and accurately decode UUU and UUC codons. Conversely, the tRNA^Ser^_AAA_ variant we investigated has adenine at 34 and 37, so it is possible that the strong reduction in protein synthesis we observed is due in part to suboptimal codon:anticodon recognition kinetics in the tRNA^Ser^_AAA_ variant compared to tRNA^Phe^.

In a previous study of tRNA^Ser^ anticodon variants expressed in HEK293 cells with an EGFP marker, similar to our observations, translation inhibition was recorded for different tRNA^Ser^ anticodon variants (21). For some tRNA^Ser^ variants, translation suppression was attributed to an integrated stress response involving phosphorylation of eIF2α at Ser52 (21). Phosphorylation of eIF2α at Ser52 is one mechanism to down-regulate translation initiation. Although tRNA^Ser^ with a Phe anticodon (tRNA^Ser^_AAA_) was not tested before, a mutant that decoded Ile codons with Ser showed a strong correlation between increased eIF2α phosphorylation and depressed protein synthesis (21). Like our observations, mutant tRNAs that decoded His and Lys codons with Ser showed a more muted or little response in p-eIF2α despite each tRNA mutant causing repression of growth and protein synthesis (21). Another study assayed eIF2α phosphorylation in stably transfected HEK293 cells expressing tRNA^Ser^ with Ala- and Leu-decoding anticodon mutations (40). In this case, eIF2α phosphorylation was never observed in cells expressing the Ala anticodon variant and only observed in cells expressing tRNA^Ser^ with a Leu anticodon after aging cells for 30 passages. Compared to normal cells, we did not observe a significant increase in eIF2α phosphorylation in cells expressing the tRNA^Ser^_AAA_ variant. The observation was corroborated by our experiments with the inhibitor ISRIB. Treatment of cells expressing wild-type tRNA with ISRIB increased protein synthesis, while treatment of cells expressing tRNA^Ser^_AAA_ with ISRIB did not increase protein production. Thus, the mistranslating cells were resistant to ISRIB. A previous study demonstrated that ISRIB fails to antagonize excessively high levels of p-eIF2α induced by arsenite treatment (75). Although eIF2a is phosphorylated in our mistranslating cells, their lack of response to ISRIB suggests mistranslating cells are defective in translation regulation.

Finally, we found that cells expressing the tRNA^Ser^_AAA_ variant were sensitive to treatment with the proteasome inhibitor MG132. The synthetic phenotype suggests that mistranslating cells rely critically on protein turnover and activity of the proteasome. We also found that protein turnover rates were reduced in cells expressing tRNA^Ser^_AAA,_ consistent with an increased burden on the proteasome caused by amino acid misincorporation. In conditions of nutrient deprivation and various forms of cellular stress (62), cells can also down-regulate elongation through inhibitory phosphorylation of eEF2 (76), which catalyzes ribosomal translocation and repositioning of tRNA from the A-site to P-site of the ribosome (77). While mistranslating cells responded to proteasome inhibition (MG132) by phosphorylated eEF2 similarly to normal cells, expression of tRNA^Ser^_AAA_ showed a dominant effect on repressing protein synthesis, regardless of stress conditions tested or eEF2 phosphorylation status. Stressors, including MG132, may also alter the activity of the mutant tRNA or the tRNome more broadly, as transcription (78) and modification (79) of tRNAs are regulated in response to stress. Investigating the interaction between mistranslating tRNAs and the impact of tRNA processing on protein quality control will form the basis of future investigations.

## CONCLUSION

Humans encode over 600 tRNA genes. Human cells and tissues are estimated to express between 200 and 400 different tRNA genes (23). While some of these genes may be redundant in function, others are critical for maintaining protein homeostasis. Despite a vast background of tRNA genes, even a single tRNA mutant has the potential to cause amino acid mis-incorporation, thus exacerbating protein misfolding diseases at the molecular level. We found that a tRNA^Ser^_AAA_ gene that occurs in 1.8% of the population indeed directs Ser mis-incorporation at Phe codons and leads to increased cytotoxicity and increased need for protein degradation in mammalian cells. Notably, mistranslating cells exhibit severely inhibited protein synthesis, leading to reduced but effective formation of protein aggregates in cellular models of HD. The mistranslating cells were also defective in clearing huntingtin aggregates and were resistant to the neuroprotective compound ISRIB. Because this compound shows promise as a treatment for neurodegeneration (80), our studies suggest that an active mistranslating tRNA in a patient's genetic background may contribute to drug resistance. Taken together, our data show that naturally occurring tRNA mistranslators have significant potential to disrupt protein homeostasis and act as modifiers of neurodegenerative disease.

## DATA AVAILABILITY

Custom imageJ macros are available in the Supplementary Information. For western blots, full size images are included as a supplementary data file. The mass spectrometry data have been deposited to the ProteomeXchange Consortium via the PRIDE (81) repository with the identifier PXD027837 (doi 10.6019/PXD027837).

## Supplementary Material

gkab898_Supplemental_Files
