# Supplementary material for: Formation and persistence of polyglutamine aggregates in mistranslating cells
Source: Nucleic Acids Res. 2021 Oct 28;49(20):11883–99. doi: 10.1093/nar/gkab898 (PMC8599886; doi:10.1093/nar/gkab898)
Supplement: gkab898_Supplemental_Files [file gkab898_supplemental_files.zip › Lant-etal-2021_SI-NAR.R1a.pdf]

Supplemental information for:

Formation and persistence of polyglutamine aggregates in mistranslating cells

Jeremy T. Lant[1], Rashmi Kiri[1], Martin L. Duennwald[3] & Patrick O'Donoghue[1,2,*].

[1]Department of Biochemistry, [2]Department of Chemistry, [3]Department of Anatomy & Cell Biology, The University of Western Ontario, London, Ontario N6A 5C1, Canada.

*Correspondence: patrick.odonoghue@uwo.ca

Supplemental Methods

Plasmids and strains. Plasmid manipulations were performed with *Escherichia coli* DH5α cells (Invitrogen). Multiple vectors for HTTexon1 expression were used. The polyQ regions were encoded by mixed CAG/CAA codon repeats. For the pcDNA3.1-derived HTTexon1 (MATLEKLMKAFESLKSF-[polyQ]-P_{11}-QLPQPP) expression plasmids, we subcloned the HTTexon1 fragment into pCDNA3.1 from a plasmid that as a kind gift of Leslie Thompson (UC Irvine) (1). The pEGFP-derived HTTexon1 (MKAFESLKSF-[polyQ]-P_{11}-QLPQPP) expression plasmids were purchased from Addgene (WT-Pan #99638, pEGFP-Q23 #40261, pEGFP-Q74 #40262). $tRNA^{Pro}$ WT and G3:U70 variants were expressed from a U6 promoter with polythymidine terminator as previously described (2). Human $tRNA^{Ser}$ genes (Ser-AGA-2-3, Ser-CGA-2-1) were PCR amplified from HEK293 genomic DNA with ~300 bp flanking sequence. Anticodon variants were introduced in PCR fragments using overlap extension PCR. tRNA expression cassettes inserted at the PciI (New England Biolabs (NEB), Ipswich, MA, USA) restriction site in pWTPAN, pEGFP-Q23, or pEGFP-Q74-derived plasmids. In pWTPAN constructs, which contain mCherry linked to EGFP, the EGFP was reverted to GFP by site-directed mutagenesis: L64F(TTC), T65S(TCT). Previously, we successfully used a EGFP D129P mistranslation sensitive reporter to observe mis-incorporation resulting from the $tRNA^{Pro}$ G3:U70 variant, that rescued fluorescence by incorporation of Ala at residue 129 (2). S65 in GFP was mutated to Phe (TTT) or Pro (CCA) with the intent of generating similar reporters sensitive to mistranslation of UUU or CCA codons with serine. We found wildtype GFP fluorescence (compared to EGFP) was insufficient to detect mistranslation by fluorescence restoration from these $tRNA^{Ser}$ variants tested. These constructs were used to track transfection efficiency of tRNA expression cassettes and measure protein synthesis rates according to the mCherry fluorescence or by Western blotting. Plasmid DNA for transfection in mammalian cells was purified by Midi-Prep (GeneAid) from 100 ml *E. coli* DH5α cultures grown at 37°C for 16 hrs to an $OD_{600} > 1.0$. DNA concentrations were measured using a Nanodrop 2000C (ThermoFisher Scientific).

Western blotting. Cells were lifted in phosphate buffered saline (1 × PBS pH 7.4; Corning Cellgro, Corning, NY, USA) supplemented with 1 mM EDTA, harvested by pipetting, and centrifuged in 1.5 ml microcentrifuge tubes at 900 × g for 3 min at 4°C. Supernatant was removed and cells washed with ice cold PBS (Corning Cellgro) and centrifuged again. Supernatant was removed and cells were suspended in 50 µl of ice-cold lysis buffer: 50 mM Tris-HCl (pH 7.4), 1% Triton X-100, 150 mM NaCl, 0.1% sodium dodecyl sulfate (SDS), and 1 mM phenylmethylsulfonyl fluoride. The re-suspended cells were incubated on ice for 5 min then centrifuged at 4°C, 20,000 × g for 10 minutes. Supernatant was collected and used immediately or flash-frozen and stored at -80°C. Protein concentrations were measured in

duplicate using the bicinchoninic acid (BCA) assay (ThermoFisher Scientific) and diluted to equimolar protein concentrations before SDS-polyacrylamide gel electrophoresis (SDS-PAGE). Lysates were separated on SDS-PAGE (10% acrylamide) with protein standards (BioRad, Hercules, CA, USA) for size determination. After SDS-PAGE, proteins were transferred to polyvinylidene fluoride membranes using a Trans-Blot Turbo Transfer System (max 25 V, 2.5 A constant for 7 min; BioRad). Membranes were incubated for 1 hr in blocking solution (3% bovine serum albumin (BSA), 0.1% Tween 20, 1% PBS) before adding primary antibodies at a 1:5000 (α-GFP, Abcam, ab32146; α-GAPDH, Sigma-Aldrich, MAB374m, α-HSP70, Invitrogen, MA3-006; α-HSP90, Protein Tech, Rosemont, IL, USA, 13171-1-AP) or 1:1000 (α-Phospho-eIF2a Ser52, ThermoFisher Scientific, 44-728G; α-eIF2a, ThermoFisher Scientific, AHO0802). Membranes were incubated with primary antibody in blocking solution overnight at 4°C, washed 3 × 10 min in washing solution (1% BSA, 0.1% Tween 20, 1% PBS), then incubated with anti-mouse (Thermo Fisher Scientific, MA1-21315) or anti-rabbit (Sigma, GENA9340) horse radish peroxidase-linked secondary antibodies for 2 hr at room temperature (~22°C) with a 1:2000 final dilution. Membranes were then washed with 1 × PBS with 0.1% Tween 20 for 3 × 10 min, followed by one wash for 10 minutes in 1 × PBS. Proteins were visualized using Clarity Western enhanced chemiluminescence (ECL) Substrates (Bio-Rad) following the manufacturer's instructions and imaged with a ChemiDoc XRS+ System (Bio-Rad).

Mass Spectrometry. mCherry protein was immunoprecipitated from N2a cells expressing wildtype $tRNA^{Ser}_{AGA}$ or mistranslating $tRNA^{Ser}_{AAA}$ after 48 hr transfection. Three 10 cm plates of transfected cells were harvested for each plasmid and lysed in ice-cold lysis buffer (see Western Blotting). mCherry protein was immunoprecipitated using RFP-trap agarose affinity resin (Chromotek, Munich, Germany) following the manufacturer's instructions. In the final step, mCherry protein was eluted by boiling the affinity beads in SDS loading dye at 95°C for 5 min. Beads were pelleted in a quickspin mini centrifuge, and supernatants were loaded on a 12% SDS-PAGE gel. Gels were stained with Coomassie blue to visualize the mCherry protein.

Bands corresponding to mCherry protein were picked from the SDS-PAGE gel using an Ettan Robotic Spot-Picker and submitted for proteolytic digestion (Trypsin) and peptide extraction at the Functional Proteomics Facility at the University of Western Ontario, Canada. Gel plugs were de-stained with 50 mM ammonium bicarbonate and 50% acetonitrile solution, dehydrated in 100% acetonitrile solution, then reduced with 10 mM DTT at 40°C. Alkylation was performed with 55 mM iodoacetamide at 40°C. Gel plugs were then washed once with 100% acetonitrile, once with 50 mM ammonium bicarbonate and 50% acetonitrile, then twice with 100% acetonitrile. Gel-bound proteins were digested overnight with 4 ng/µl trypsin in 50 mM ammonium bicarbonate at 40°C. Trypsinized proteins were extracted from gel plugs with 2% formic acid and 2% acetonitrile.

The dried sample was reconstituted in 40 µl for sample WT, and in 25 µl or sample VAR, of 0.1% Formic acid in water and 1 µl was injected onto an ACQUITY MClass UPLC system using an ACQUITY UPLC MClass Symmetry C18 trap column (Waters Corporation, Milford, MA), at a flow rate of 5 µl/min for 6 minutes using 99% buffer A (0.1% formic acid) and 1% buffer B (Acetonitrile + 0.1% formic acid). After trapping the peptides were eluted onto the analytical column for separation, using a 90 min run time. Flow was established at 300 nl/min for the ACQUITY UPLC MClass Peptide BEH C18 Column 15K psi, 130A, 1.7 µm x 25mm which was held at 35°C. The gradient initial condition was 1% buffer B. Buffer B then increased to 7.0% over 1 min, then to 23% over 44 min, then to 35% over 15 min, then to 98% over 5 min, then held at 98% for 5 min, before washing and re-equilibration steps. The LC system was directly connected to a NanoFlex (Thermo Electron Corp., Waltham, MA) nanospray ionization source with a

source voltage of 2.3 KV and was interfaced to a QExactive Plus mass spectrometer (Thermo Electron Corp., Waltham, MA). The mass spectrometer was controlled by Xcalibur software (Thermo, v. 2.8.1) and operated in the data-dependent mode using an FT/FT/HCD Top 12 scheme. The MS scan recorded the mass-to-charge ratios (*m*/*z*) of ions over the range of 375–1500 with a resolution of 70,000 at m/z 400, positive ion, profile, full MS mode using a lock mass (445.120025 m/z). The 12 most abundant multiply charged ions were automatically selected for subsequent high energy collisional induced dissociation in the HCD cell, (FT/HCD) with an isolation width of 2.00 Da, and a 0.5 Da offset, centroid mode, 17500 resolution in the orbitrap, with charge state filtering allowing only ions of +2, to +6 charged states. Normalized Collision energy was 25, and precursor ions were then excluded from further HCD for 15s.

Raw data files were loaded, processed, and searched using Peaks X+ (Bioinformatics Solutions Inc.), against a custom database, consisting of a group of common contaminants and possible proteoforms of mCherry resulting from Phe-to-Ser, and Leu-to-Ser possible substitutions. An FDR of 0.1% and at least 1 unique peptide were used. A maximum of 3 missed cleavages (MC) were allowed for searching tryptic peptides. Fixed modification of carbamidomethylation (CAM) Cysteine, and variable modifications of deamidation (N/Q), oxidation (M, H, W), sulphone (M), and iodination (YH) with a parent mass error tolerance of 10.0 ppm and fragment mass error tolerance of 0.02 Da were used, allowing non-specific cleavage at one end of peptide. Hits representing Phe-to-Ser misincorporation were curated to include only peptides with area-under-the-curve values ≥ $1x10^7$ and with y and b ion spectra capturing the misincorporation event. Example peptides demonstrating mis-incorporation (Table 2, Fig. S1) as well as a complete list (Table 3) are included.

Fluorescence microscopy. ImageJ macros are included in the appendix of this file with detailed analysis, descriptions, and line-by-line commentary. Fluorescent microscopy images were captured on an EVOS FL auto fluorescent microscope (Thermo Fisher Scientific). GFP (470 ± 22 nm excitation, 510 ± 42 nm emission) and RFP (531 ± 40 nm excitation, 593 ± 40 nm emission) filter cubes were used to capture green or red fluorescence. In our initial experiments (Fig. 2A), fluorescence was quantitated on the EVOS software by drawing ellipses within fluorescing cells as described previously (2). For subsequent fluorescence analyses, we used semi-automated ImageJ macros to annotate cells and measure intensity within regions of interest (ROI; see appendix). For experiments with ISRIB, 500nM ISRIB dissolved in DMSO or an equivalent volume of DMSO was added to cells after 24 hrs transfection. Images were captured immediately after adding ISRIB and again after 18 hr incubation. For fluorescence-based aggregation experiments, aggregates of EGFP-fused polyQ proteins were visible after 24-48 hrs, and all images of aggregates were captured at least 24 hrs post-transfection unless otherwise stated. To quantify insoluble protein aggregate size, an established membrane detergent assay with Triton X-100 was performed (3). Initial images were captured 48 hrs post-transfection, cells were incubated for 40 min in media containing 0.25% Triton X-100, and images were captured again from the same location using the EVOS microscope. The size of the insoluble aggregates was quantified using ImageJ thresholding analysis (see appendix) similarly to published approaches (4). Scale bars were used to determine the conversion factor of pixels to μm^2. For live cell imaging, cells were incubated at 37°C with humidity and 5% CO_2 in the EVOS FL auto-fluorescent microscope environment chamber. Images were captured every 30 minutes for an 18 hr time-course, starting 24 hrs post-transfection. Fluorescence and number of aggregates were quantified using a semi-automated approach in ImageJ (see appendix) based on established approaches to quantitate protein aggregation (4).

Supplemental Tables

Table S1. Oligonucleotide sequences

Description	Nucleotide sequence
tRNA-Ser-AGA-2-3 genomic PCR (Fwd)	CCTGGAAGTCCGAACACC
tRNA-Ser-AGA-2-3 genomic PCR (Rev)	GTGAACACAAAGATGAGAGACACC
tRNA-Ser-AGA-2-3 nested PCR with PciI cloning site (Fwd)	CAGACTACATGTGTTGGCCATGACTCCCC
tRNA-Ser-AGA-2-3 nested PCR with PciI cloning site (Rev)	CAGACTACATGTGCAATTCCGTGAGGGAAATTCG
tRNA-Ser-AGA-2-3 G35A mutagenesis (Fwd)	GCGATGGACTAAAAATCCATTGGGGTCTCCCC
tRNA-Ser-AGA-2-3 G35A mutagenesis (Rev)	CCAATGGATTTTTAGTCCATCGCCTTAACCACTC
tRNA-Ser-CGA-2-1 nested PCR with PciI cloning site (Fwd)	CAGACTACATGTACATGCACAGCAGCGTTC
tRNA-Ser-CGA-2-1 nested PCR with PciI cloning site (Rev)	CAGACTACATGTTTGCCGTTAGAATCTGTCGC
tRNA-Ser-CGA-2-1 C34T, A36G mutagenesis (Fwd)	CGTTGGACTTGGAATCCAATGGGGTCTCCCC
tRNA-Ser-CGA-2-1 C34T, A36G mutagenesis (Rev)	CCATTGGATTCCAAGTCCAACGCCTTAACCAC
Replace EGFP in WT-PAN with GFP mutant with EcoRI (Fwd)	CAGACTGAATTCGCCACCATGGTGAGCAAG
Replace EGFP in WT-PAN with GFP mutant with BamHI (Fwd)	CAGACTGGATCCGACTTGTACAGCTCGTCCATG
GFP S65F(UUU) mutagenesis (Fwd)	GACCACCTTCTTTTACGGCGTGCAGTGCTTC
GFP S65F(UUU) mutagenesis (Rev)	GCCGTAAAAGAAGGTGGTCACGAGGGTGG
GFP S65P(CCA) mutagenesis (Fwd)	GACCACCTTCCCATACGGCGTGCAGTGCTTC
GFP S65P(CCA) mutagenesis (Rev)	GCCGTATGGGAAGGTGGTCACGAGGGTGG

Table S2. tRNA gene sequences and identifiers

tRNA gene[a]	genomic sequence[b]	locus	variants	dbSNP ID[c]
Ser-AGA-2-3	GTAGTCGTGGCCGAGTGGTTAAGGCGATGGACTAGAAATCC ATTGGGGTCTCCCCGCGCAGGTTCGAATCCTGCCGACTACG	chr6:27463593-27463674	G35A	rs147439337
Ser-CGA-2-1	GCTGTGATGGCCGAGTGGTTAAGGCGTTGGACTCGAAATCC AATGGGGTCTCCCCGCGCAGGTTCAAATCCTGCTCACAGCG	chr6:27177628-27177709	C34T, A36G	n.a.[d]
Pro-TGG-1-1	GGCTCGTTGGTCTAGTGGTATGATTCTCGCTTTGGGTGCGA GAGGtCCCGGGTTCAAATCCCGGACGAGCCC	chr14:21101165-21101236	C3G, G70T	n.a.[d]

[a]GtRNAdb gene symbol from high confidence tRNA gene set (*Human Feb. 2009 GRCh37/hg19*) (5). [b]None of the listed tRNA genes contain introns compared to the predicted mature tRNA sequence (5). [c]dbSNP is the NCBI database of genetic variation (build 155; released Apr. 9, 2021) (6). [d]Synthetic variants, no associated SNP ID.

Supplemental Figures

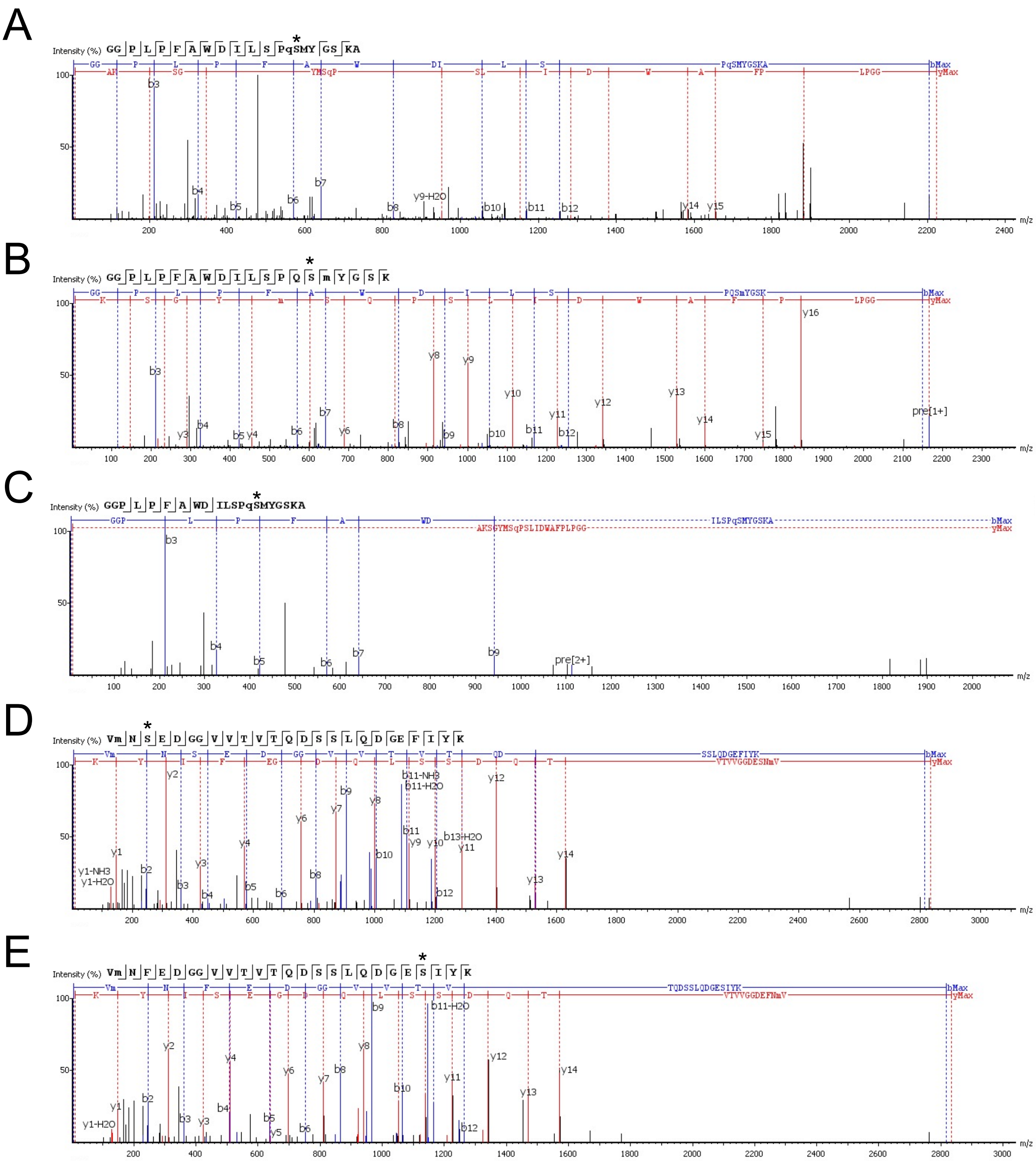

Figure S1. Detailed y and b ion spectra of observed Ser mistranslation at Phe codons. MS/MS spectra of high abundance peptides (area > 1×10^{7}) described in Table 2 are shown. Stars indicate Phe (UUC) codons that were mistranslated as Ser. One low scoring peptide hit (-10logP = 58.8) was observed from cells expressing (A) $tRNA^{Ser}_{AGA}$ and a (C) similar low scoring hit (-10logP = 48) was found in mistranslating cells. (B, D, E) Multiple high-quality hits (-10logP > 88) demonstrated Ser mis-incorporation at three different Phe codons in mCherry in cells expressing $tRNA^{Ser}_{AAA}$.

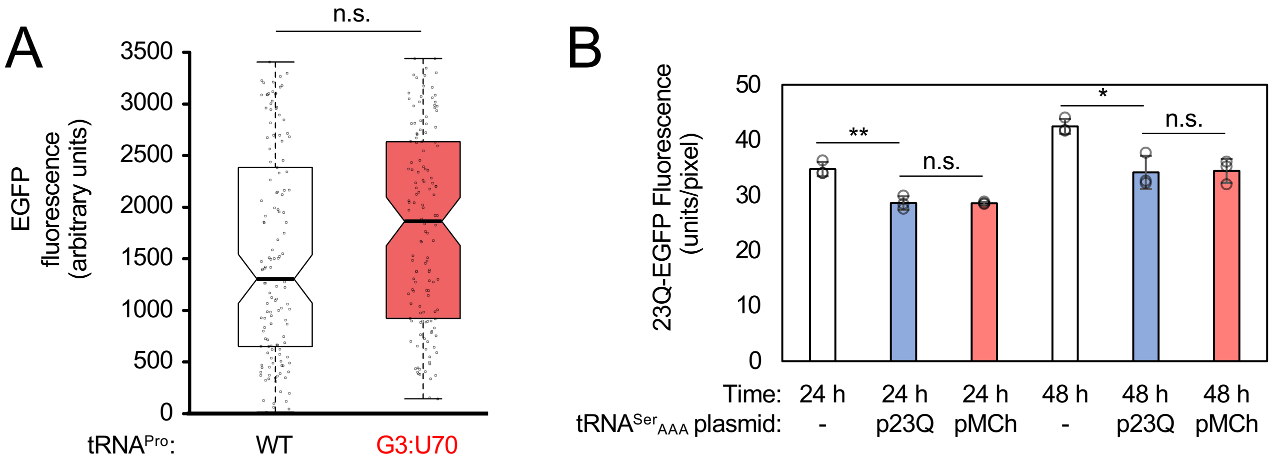

Figure S2. HEK293 cells expressing tRNA^{Pro} G3:U70 and cis/trans-plasmid expression tests of N2a cells expressing tRNA$^{Ser}_{AAA}$. (A) HEK293 cells were transfected with a plasmid encoding human tRNA^{Pro} or the tRNA^{Pro} G3:U70 variant and wild-type EGFP as previously described (2). Fluorescence of cells was measured by fluorescence microscopy in GFP settings. Significant differences (independent sample t-test) of biological means are marked (n.s. = no significant difference). (B) N2a cells were co-transfected with two plasmids: one encoding 23Q-EGFP (p23Q) and another encoding mCherry (pMCh). The tRNA$^{Ser}_{AAA}$ variant was either not included (-) or cloned into the p23Q or the pMCh plasmid as indicated. Cellular fluorescence was captured by fluorescence microscopy in GFP and RFP settings at 24 hr and 48 hr timepoints. Fluorescence per unit area was quantitated in ImageJ. Error bars represent the mean ± 1 standard deviation of three biological replicates; stars indicate statistically significant differences according to independent sample t-tests (n.s. = no significant difference, * = $p < 0.05$, ** = $p < 0.01$).

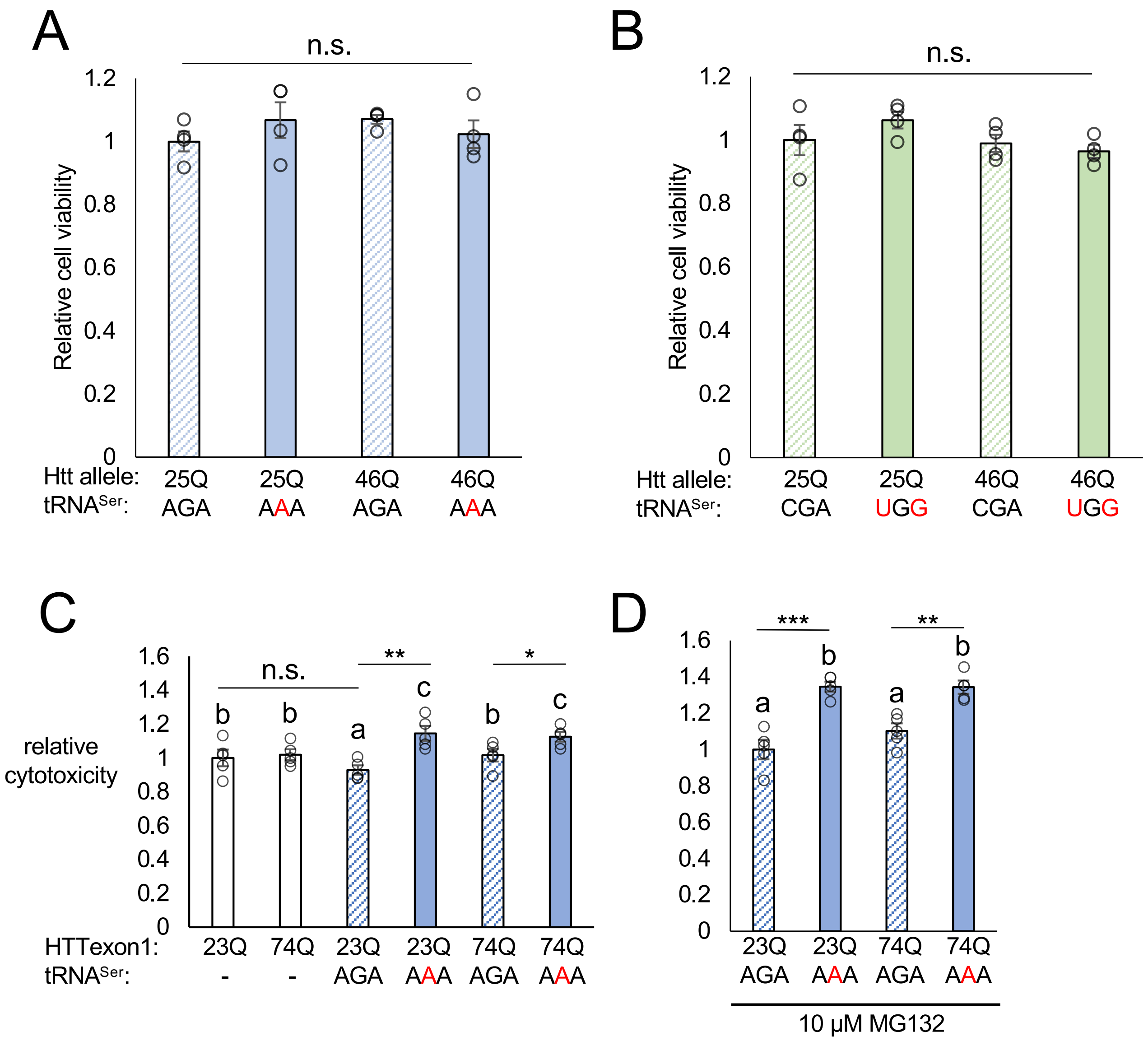

Figure S3. Viability of cells mistranslating Phe or Pro codons with Ser and expressing HTTexon1 variants. (A, B) N2a cells were co-transfected with two plasmids encoding HTTexon1 25Q or 46Q and (A) $tRNA^{Ser}_{AGA}$ or G35A variant $tRNA^{Ser}_{AAA}$ or (B) $tRNA^{Ser}_{CGA}$ or C34G, A36G variant $tRNA^{Ser}_{UGG}$. Cellular viability was assayed 24 hr post-transfection with the CellTitreGlo 2.0 assay. Luminescence readings were normalized to the (A) 25Q/AGA or (B) 25Q/CGA controls. (C, D) N2a cells were transfected with a plasmid encoding human $tRNA^{Ser}_{AGA}$ or G35A variant $tRNA^{Ser}_{AAA}$ and HTTexon1 containing 23Q- or 74Q-EGFP. (C) Cytotoxicity was assayed 48 hr post-transfection with the CytotoxGlo assay. (D) The experiment was repeated with a 4-hour treatment of 10μM MG132 before assay. Luminescence readings were normalized to the (C) 23Q/no tRNA control or to the (D) 23Q/AGA control. Stars represent p-values from (A,B) ANOVA or (C,D) pairwise independent sample t-tests (n.s. = no significant difference, * = $p < 0.05$, ** = $p < 0.01$, *** = $p < 0.001$); letters represent significantly different groups from Tukey's honestly significant difference tests ($\alpha = 0.05$). Each bar represents the mean ± 1 standard deviation of four (A,B) or five (C,D) biological replicates.

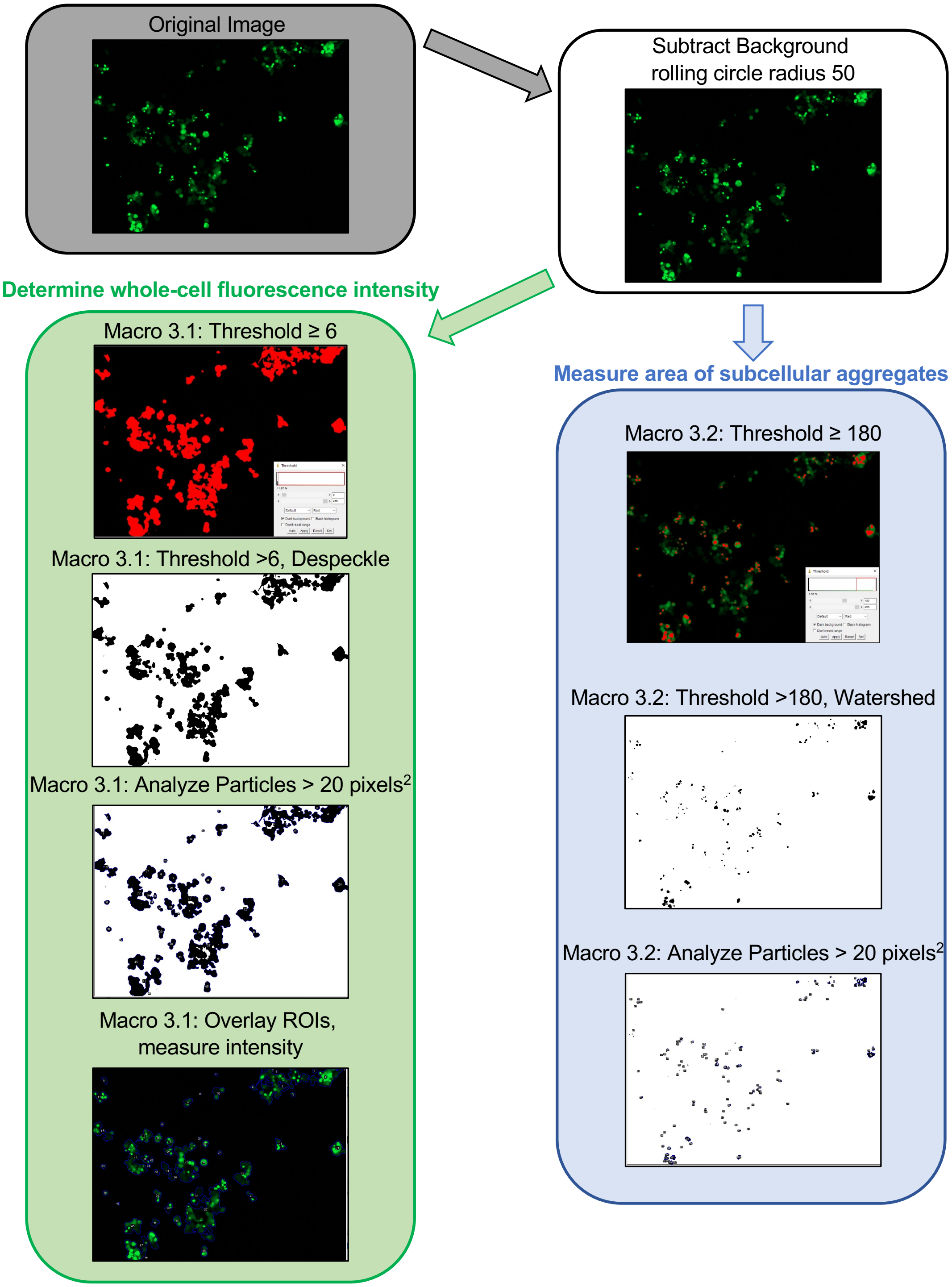

Figure S4. Quantitation of aggregate fluorescence and area in ImageJ. Images represent steps in ImageJ macros used to quantitate fluorescent aggregates in live cell data shown in Fig. 4. See supplemental methods and appendix for details and ImageJ macros.

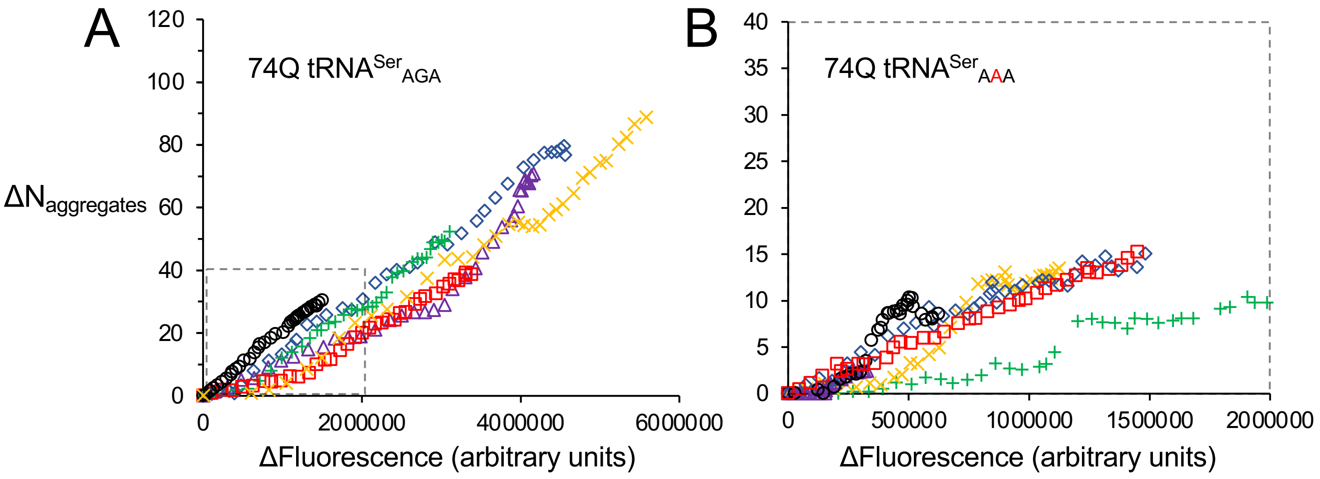

Figure S5. Live cell imaging raw fluorescence and number of aggregates ($N_{aggregates}$). Raw data from live cell imaging experiment shown in Fig. 4B. The data represent replicates showing the relationship between ΔFluorescence and $\Delta N_{aggregates}$, where each point is a single 30 min timepoint in one field of view. Images were captured for 18 hr as described in Fig. 4 (see also Fig. S4 and supplementary information appendix).

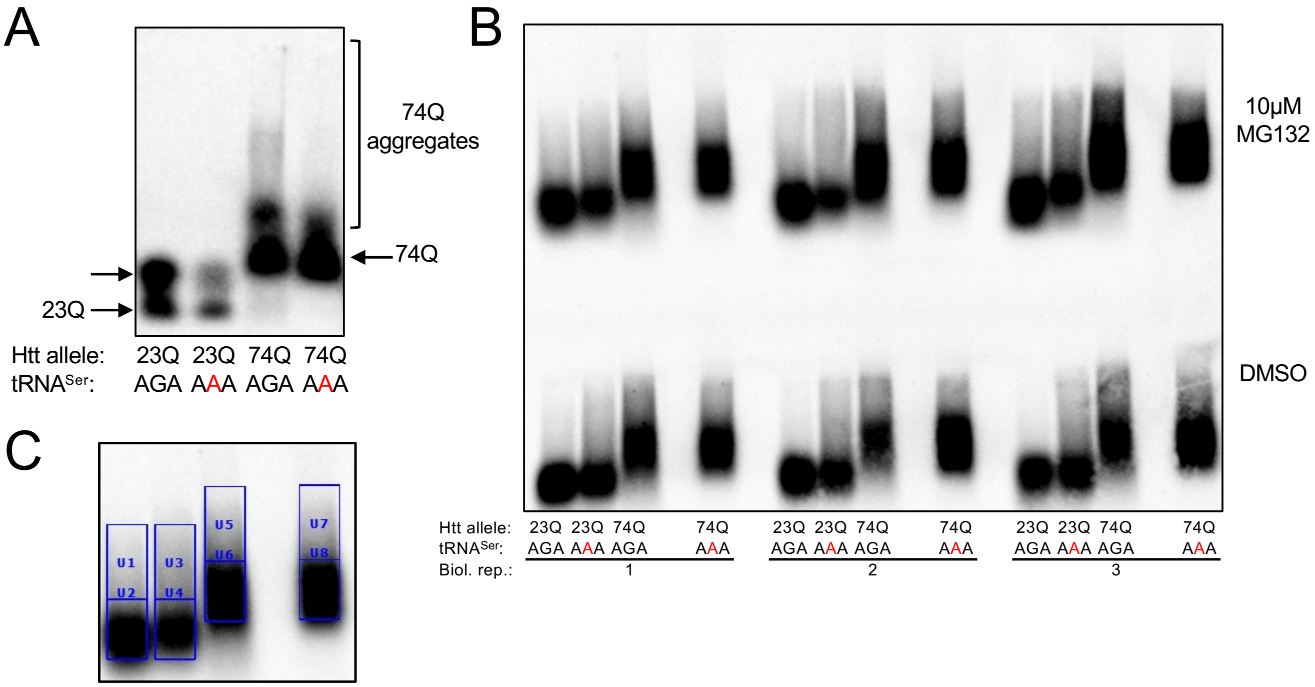

Figure S6. Semi-denaturing detergent acrylamide gel electrophoresis (SDD-AGE). N2a cells were transfected with a plasmid encoding human $tRNA^{Ser}_{AGA}$ or G35A variant $tRNA^{Ser}_{AAA}$ and HTTexon1 containing 23Q- or 74Q-EGFP. Cell lysates were harvested and analyzed by SDD-AGE and western blotting (αGFP) after 24 hr (A) or 48 hr (B) transfection. Higher molecular weight smears in the 74Q lanes indicate the presence of aggregated proteins. Cells in (B) were treated for 4 hr in DMSO or 10 μM MG132 prior to lysis. Blue boxes (C) were used for densitometry quantitation shown in Fig. 5D.

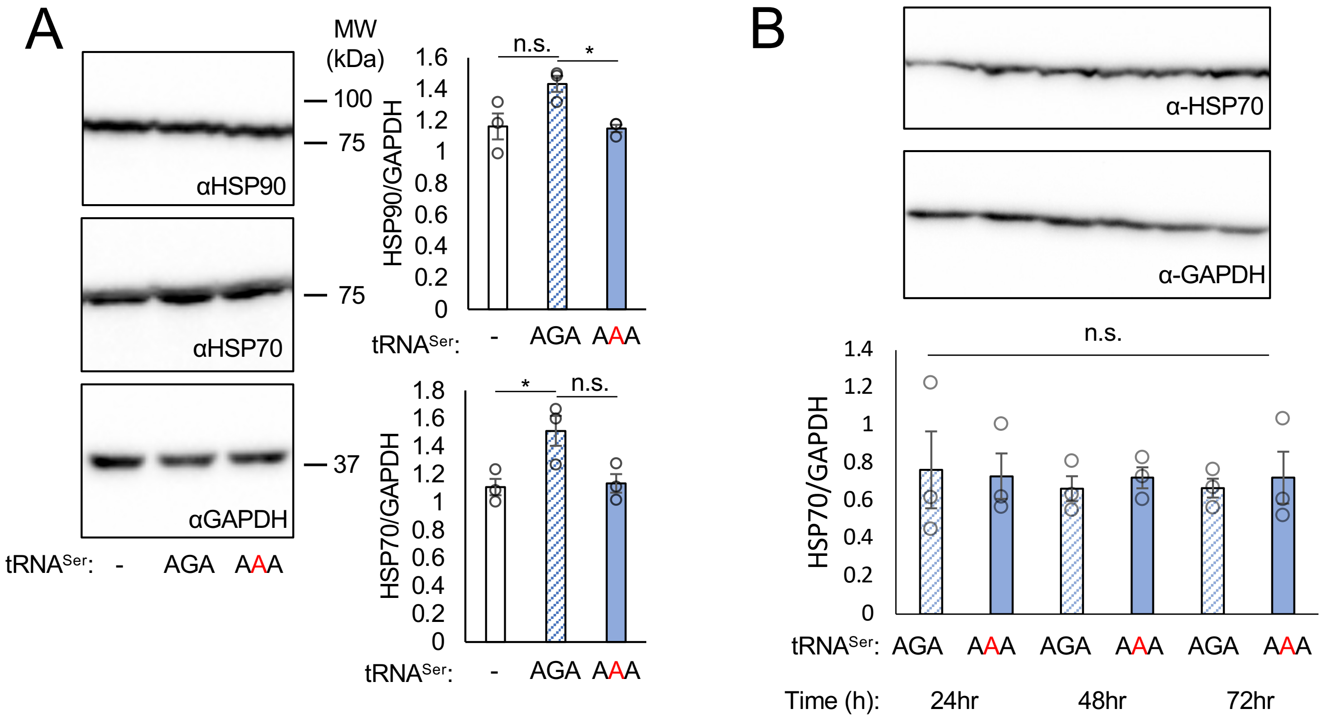

Figure S7. Heat shock protein levels in mistranslating cells. N2a cells were transfected with a plasmid encoding human $tRNA^{Ser}_{AGA}$ or G35A variant $tRNA^{Ser}_{AAA}$ and dead GFP(S65F)-mCherry transfection marker. (A) After 24 hr transfections, cell lysates were harvested and western blotted for HSP90, HSP70, and GAPDH loading control. (B) The same transfections were repeated with longer transfection periods and western blotted for HSP70 and GAPDH. Significant differences (independent sample t-test) of three biological replicates are marked with stars (n.s. = no significant difference, * = $p < 0.05$). Error bars represent ± 1 standard deviation.

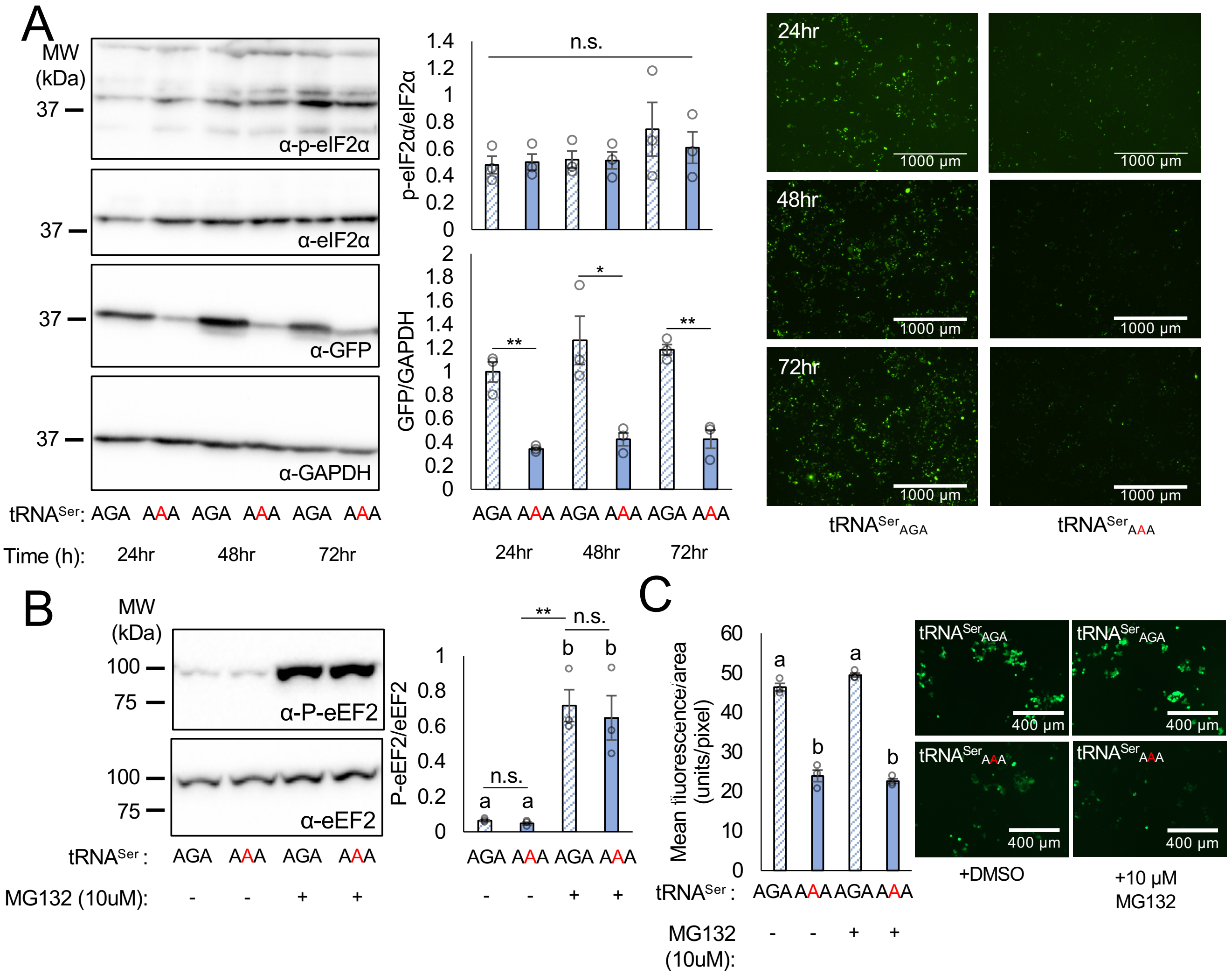

Figure S8. Regulation of translation initiation and elongation in mistranslating cells. (A) N2a cells were transfected with a plasmid encoding human $tRNA^{Ser}_{AGA}$ or G35A variant $tRNA^{Ser}_{AAA}$ and HTTexon1 containing 23 (23Q) CAG/CAA mixed codon repeats encoding polyQ fused to EGFP as a transfection marker. Cells were imaged by fluorescence microscopy in GFP settings (A, right panel), harvested, lysed, and western blotted with anti-p-eIF2*α* (pSer52), anti-eIF2*α*, anti-GFP, and anti-GAPDH antibodies after 24, 48, or 72 hr transfection. (B) N2a cells were transfected with a plasmid encoding human $tRNA^{Ser}_{AGA}$ or G35A variant $tRNA^{Ser}_{AAA}$ and mCherry for 48 hr, treated with MG132 or DMSO for 4 hr, and western blotted with anti-eEF2, anti-p-eEF2 (pThr56). (C) N2a cells were transfected as in (A) and fluorescence was measured after 4 hr treatment with DMSO or MG132. Stars indicate p-values from independent sample t-tests (n.s. = no significant difference, * = $p < 0.05$, ** = $p < 0.01$) and letters indicate significantly different groups determined by Tukey's HSD test ($\alpha = 0.05$).

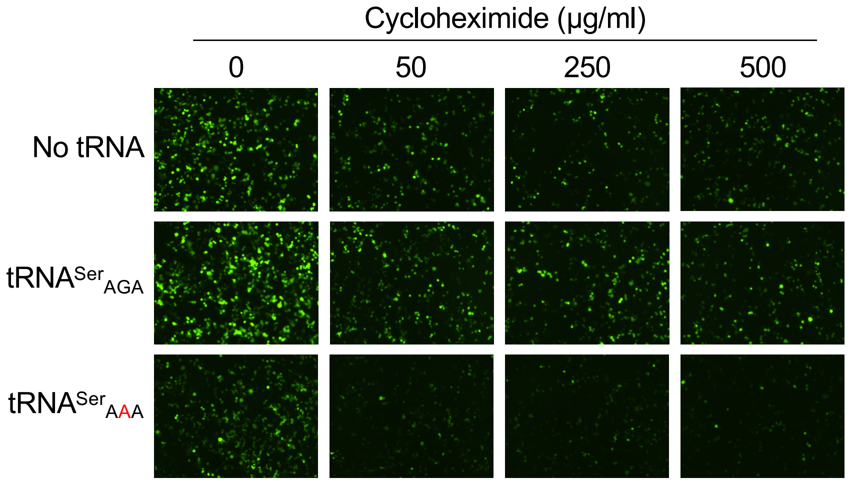

Figure S9. Cycloheximide concentration testing. N2a cells were transfected with a plasmid encoding human $tRNA^{Ser}_{AGA}$ or G35A variant $tRNA^{Ser}_{AAA}$ and HTTexon1 containing 23Q- or 74Q-EGFP for 48 hr. Cells were washed and treated with indicated concentrations of cycloheximide for 24 hr before capturing images by fluorescence microscopy in GFP settings.

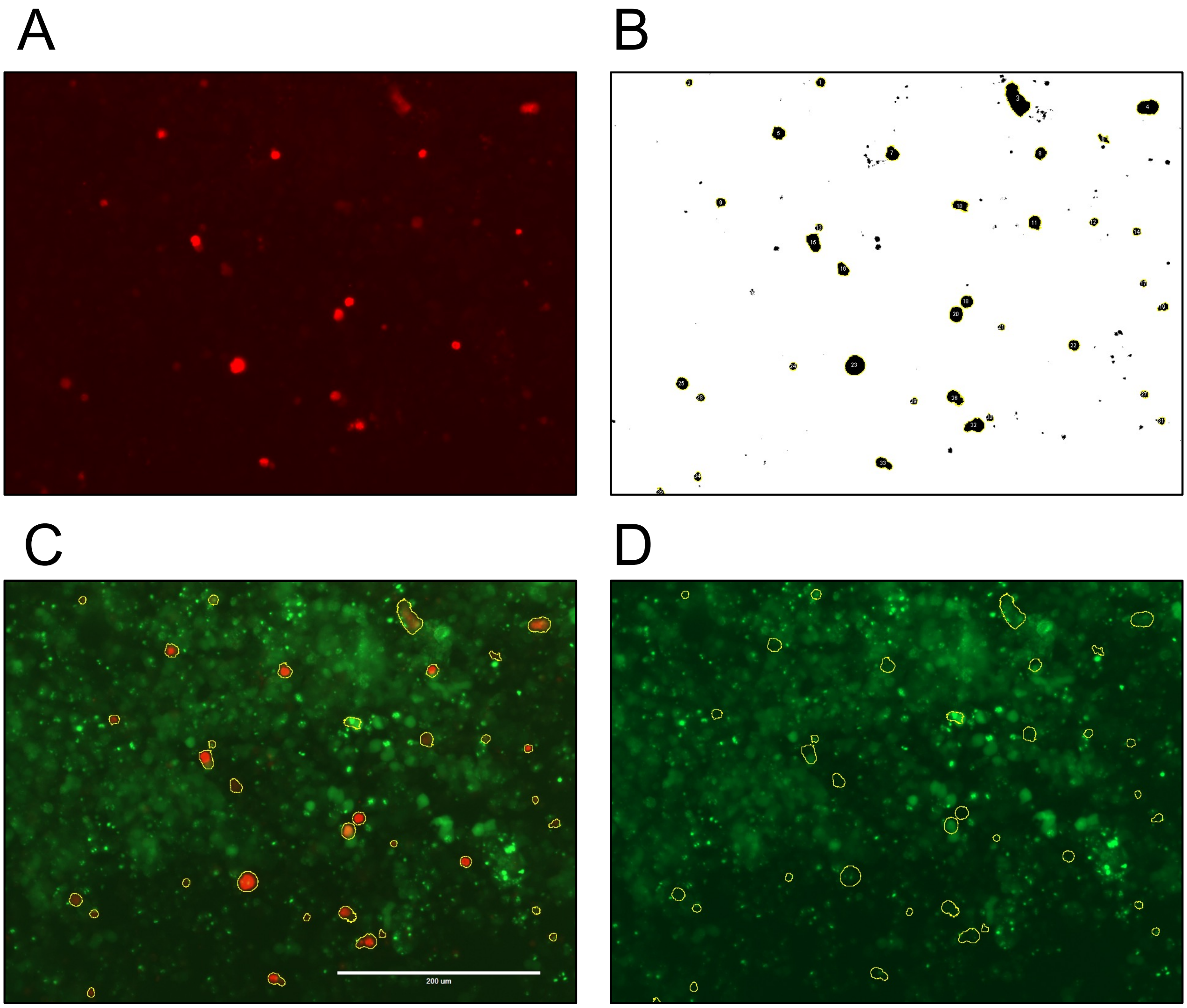

Figure S10. Aggregate counting in PC12 cells transfected with mCherry-tRNA plasmids. Mistranslating $tRNA^{Ser}_{AAA}$ and wildtype $tRNA^{Ser}_{AGA}$ were co-expressed with mCherry on plasmids to identify transfected cells. (A) Red channel images were used to generate region of interest markers in ImageJ using (B) threshold and analyze particles functions, then overlayed on (C,D) green channel images to identify cells containing the ectopically expressed tRNA variant and EGFP-tagged polyQ aggregates.

Supporting Data File

Data File S1. Single cell kinetics of polyQ formation in wild type and mistranslating cells. Image series from live cell fluorescence experiments described in Fig. 4 were imported to ImageJ and background fluorescence was subtracted using the rolling circle function. Image series were converted to surface plots using the analyze; surface plot function and saved as .gif files. Peaks represent fluorescent objects in a single field of view. Peak heights represent brightness intensity on a 0-255 scale where zero is black and 255 is maximum pixel intensity.

Supplemental Appendix: Macros

ImageJ macros and analyses. The following text describes Fiji/ImageJ (7,8) macros used in the manuscript. ImageJ version 1.53c was run on a Windows 10 (64-bit) operating system. Lines beginning with // are commentary describing the line below. See Fig. S4 for step-by-step example images showing Macros 1.1-1.2. Other macros use a similar thresholding approach.

Macros 1.1 and 1.2: Live cell fluorescence and aggregate count. Live cell image analysis (Fig. 4) was done with two split macros on the same image sequences. See Fig. S4 for images demonstrating the method. Data sets obtained from ImageJ macros were processed and analyzed in Microsoft Excel. Each image (Fig. 4) represented a single time point in one field of view. For each treatment (WT or mutant tRNA), two fields of view were used on three biological replicates, totaling six technical replicates. For fluorescence analysis, image numbers paired to ROIs were copied from the ImageJ ROI manager along with integrated density values from the results window (Macro 1.1 and Macro 1.2). Using Microsoft Excel, total fluorescence contained within ROIs was calculated for each image (cellular fluorescence).

For aggregate analysis, image numbers paired to ROIs were copied from the ImageJ ROI manager along with area values from the results window (Macro 1.2). Using Microsoft Excel, we calculated the sum of area covered by pixels over our aggregation threshold (brightness and size) for each image. We then calculated the average aggregate size across all images. The sum of area covered by aggregates in each image was divided by average aggregate size to estimate the number of aggregates in each image. This approach corrects for clustered objects that cannot be separated by the watershed function. Similar approaches have been used by others to count fluorescent polyQ protein aggregates in cells (4). For each image sequence (technical replicate), the estimated number of aggregates was plotted against fluorescence (Fig. S5). Initial values were subtracted from all data points in each series to exclude fluorescence and aggregates present before the beginning of the time course. Number of aggregates was normalized to image fluorescence for each image series and plotted against time (Fig. 4). Error bars represent one standard deviation of the mean for each time point.

Macro 1.1.
This macro is to select area containing fluorescing cells from a series of fluorescent cell images, create region of interest (ROI) annotations, and measure fluorescence (integrated density).

```
//Import image sequence. Replace "file" with desired image series location.
"number" is the number of images in the series and "increment" is used to
exclude unwanted images in the file folder.
run("Image Sequence...", "open=[file] number=37 increment=2 sort");
```

```
//Subtract background signal using rolling circle method with radius of 50
pixels.
run("Subtract Background...", "rolling=50 stack");
//Set threshold to exclude pixels below chosen cutoff (6 in this case).
Fine-tune according to your image brightness and use same cutoff for all
images in one experiment.
setThreshold(6, 255);
//Next two lines convert image to black features (above threshold) on white
background (below threshold)
setOption("BlackBackground", false);
run("Convert to Mask", "method=Default background=Dark");
//Remove noise with despeckle function
run("Despeckle", "stack");
//Create annotations covering area above threshold. Excludes objects under
size cutoff of 20 pixels. Size cutoff should be adjusted depending on the
magnification of the image and the size of objects you wish to exclude
(noise).
run("Analyze Particles...", "size=20-Infinity display clear summarize add
stack");
//Clear results. This macro is only to create ROIs so results meaningless
at this point.
run("Clear Results");
//Import unmodified image sequence again. Should be identical to line 1
run("Image Sequence...", "open=[file] number=37 increment=2 sort");
//Subtract background signal using rolling circle method with radius of 50
pixels.
run("Subtract Background...", "rolling=50 stack");
//Set measurements to record integrated density to 3 decimals.
run("Set Measurements...", "integrated redirect=None decimal=3");
//The next three lines overlay the ROIs generated from thresholding steps
on the original image sequence, calculate the intensity within the ROIs,
and display the results in the "Results" window.
roiManager("Show None");
roiManager("Show All");
roiManager("Measure");
```

Macro 1.2.
This macro is to select area containing fluorescing protein aggregates from a series of fluorescent cell images, create region of interest (ROI) annotations, and measure area. The threshold should be fine-tuned to capture only subcellular objects (protein aggregates) and avoid whole fluorescent cells.

```
//import image sequence. Should be identical to line 1 in Macro 3.1
run("Image Sequence...", "open=[file] number=37 increment=2 sort");
//Subtract background signal using rolling circle method with radius of 50
pixels.
run("Subtract Background...", "rolling=50 stack");
//Set threshold to exclude pixels below chosen cutoff (180 in this case).
Fine-tune according to your image brightness and use same cutoff for all
images in one experiment.
setThreshold(180, 255);
//Next two lines convert image to black features (above threshold) on white
background (below threshold)
setOption("BlackBackground", false);
```

```
run("Convert to Mask", "method=Default background=Dark");
//Watershed function separates closely adjacent objects with a line. This
is important when measuring area of objects.
run("Watershed");
//Creates annotations covering area above threshold. Excludes objects under
size cutoff of 20 pixels. Size cutoff should be adjusted depending on the
magnification of the image and the size of objects you wish to exclude
(noise).
run("Analyze Particles...", "size=20-Infinity display clear summarize add
stack");
//Clear results outputting from analyze particles function
run("Clear Results");
//next two lines measure the area of objects above the intensity and size
cutoffs and display the results in the "Results" window.
run("Set Measurements...", "area redirect=None decimal=3");
roiManager("Measure");
```

Macro 2.1: Aggregate size and count. The output of this analysis includes the count and size of objects in fluorescent cell images (Fig. 5B). Nine images were captured across three biological replicates from each transfected plasmid. Images were captured before and after Triton X-100 treatment. Post-treatment images were used for size analysis. Scale bars was measured in ImageJ to calculate the conversion factor of pixels to μm^2. The area values of fluorescent objects (post-Triton X-100) outputted from ImageJ were converted from pixels to μm^2 and all data were plotted as boxplots using R. Outliers exceeding 1.5X the interquartile range of the entire dataset were omitted from plots and statistical analysis. Outlier removal excludes clustered objects that are not properly separated by the watershed function. Statistical differences were calculated with an independent sample t-test based on means of biological replicates.

Macro 2.1.

```
//open image. Replace "file" with desired image location.
open("file");
//Subtract background signal using rolling circle method with radius of 50
pixels.
run("Subtract Background...", "rolling=50");
//Set threshold to exclude pixels below chosen cutoff (100 in this case).
Fine-tune according to your image brightness and use same cutoff for all
images in one experiment.
setThreshold(100, 255);
//Next two lines convert image to black features (above threshold) on white
background (below threshold)
setOption("BlackBackground", false);
run("Convert to Mask");
//Remove noise with despeckle function
run("Despeckle");
//Watershed function separates closely adjacent objects with a line.
run("Watershed");
//Set measurements to record object area
run("Set Measurements...", "area redirect=None decimal=3");
//Record measurements and display results summary
run("Analyze Particles...", "display clear summarize add");
```

Macro 3.1: % increase fluorescence/area. The "mean" measurement parameter on ImageJ is equal to the integrated density divided by area (pixels) within a region of interest (ROI). ROI annotations were used to select only the area in images covered by fluorescing cells. Mean (fluorescence/area) was measured rather than integrated density to accommodate differences in cell sizes or cell clusters which could not be resolved by the watershed function. Two images were captured from each of three biological replicates, totaling six technical replicates. Images from before (i) and after (f) an 18 hr time course were used in analysis. "% increase fluorescence/area" (Fig. 6B) was calculated by the following formula:

$$\% \, increase \frac{fluorescence}{area} = 100 \times \left(\frac{fluorescence_i}{area_i} - \frac{fluorescence_f}{area_f} \right)$$

Statistical differences were calculated with an independent sample t-test based on means of biological replicates.

Macro 3.1.

```
//open image. Replace "file" with desired image location.
open("file");
//Subtract background signal using rolling circle method with radius of 50
pixels.
run("Subtract Background...", "rolling=50");
//Set threshold to exclude pixels below chosen cutoff (6 in this case).
Fine-tune according to your image brightness and use same cutoff for all
images in one experiment.
setThreshold(6, 255);
//Next two lines convert image to black features (above threshold) on white
background (below threshold)
setOption("BlackBackground", false);
run("Convert to Mask");
//Remove noise with despeckle function
run("Despeckle");
//Watershed function separates closely adjacent objects with a line.
run("Watershed");
//Analyze particles to generate ROI annotations. Excludes objects under
size cutoff of 20 pixels. Size cutoff should be adjusted depending on the
magnification of the image and the size of objects you wish to exclude
(noise).
run("Analyze Particles...", "size=20-Infinity display clear summarize
add");
//Clear results
run("Clear Results");
//Reopen the same file from step 1
open("file");
//Subtract background signal using rolling circle method with radius of 50
pixels.
run("Subtract Background...", "rolling=50");
//Overlay ROI markers on reopened image
roiManager("Show None");
roiManager("Show All");
//Set measurements to mean and measure
run("Set Measurements...", "mean redirect=None decimal=3");
roiManager("Measure");
```

Macro 4.1: Fluorescence/initial cell area. This macro is to select area containing fluorescing cells from a series of fluorescent cell images, create region of interest (ROI) annotations, and measure fluorescence (integrated density) and area of the ROIs. This was used to quantitate decreasing fluorescence in cycloheximide chase assays (Fig 7). A modified analysis was used in this case because cycloheximide-treated cells shrink in size over time. Hence, we found that normalizing fluorescence/area on a per-image basis was ineffective. Therefore, we instead normalized the total fluorescence in each image sequence to the area of fluorescing cells in the first image of the same sequence. We then subtracted the initial values of fluorescence/area for each sequence and calculated the average slope of each data sequence to produce the "Delta Fluorescence/Initial Cell Area" and "Average Slope" plots shown in Fig. 7. Six image sequences were captured across three biological replicates for each combination of transfected plasmid and treatment condition.

```
//Import image sequence. Replace "file" with desired image series location.
"number" is the number of images in the series and "increment" is used to
exclude unwanted images in the file folder.
run("Image Sequence...", "open=[file] number=49 starting=2 increment=3
sort");
//Subtract background signal using rolling circle method with radius of 50
pixels.
run("Subtract Background...", "rolling=50 stack");
//Set threshold to exclude pixels below chosen cutoff (6 in this case).
Fine-tune according to your image brightness and use same cutoff for all
images in one experiment.
setThreshold(10, 255);
//Next two lines convert image to black features (above threshold) on white
background (below threshold)
setOption("BlackBackground", false);
run("Convert to Mask", "method=Default background=Dark");
//Remove noise with despeckle function
run("Despeckle", "stack");
//Watershed function separates closely adjacent objects with a line.
run("Watershed", "stack");
//Analyze particles to generate ROI annotations. Excludes objects under
size cutoff of 100 pixels. Size cutoff should be adjusted depending on the
magnification of the image and the size of objects you wish to exclude
(noise).
run("Analyze Particles...", "size=100-Infinity display clear summarize add
stack");
//Clear results
run("Clear Results");
//Reopen the same file from step 1
run("Image Sequence...", "open=[file] number=49 starting=2 increment=3
sort");
//Overlay ROI markers on reopened image
roiManager("Show None");
roiManager("Show All");
//Set measurements to area and integrated density, then measure
run("Set Measurements...", "area integrated redirect=None decimal=3");
roiManager("Measure");
```

Supplemental References

1. O'Rourke, J.G., Gareau, J.R., Ochaba, J., Song, W., Rasko, T., Reverter, D., Lee, J., Monteys, A.M., Pallos, J., Mee, L. *et al.* (2013) SUMO-2 and PIAS1 modulate insoluble mutant huntingtin protein accumulation. *Cell Rep*, **4**, 362-375.
2. Lant, J.T., Berg, M.D., Sze, D.H.W., Hoffman, K.S., Akinpelu, I.C., Turk, M.A., Heinemann, I.U., Duennwald, M.L., Brandl, C.J. and O'Donoghue, P. (2018) Visualizing tRNA-dependent mistranslation in human cells. *RNA Biol*, **15**, 567-575.
3. Titus, S.A., Southall, N., Marugan, J., Austin, C.P. and Zheng, W. (2012) High-Throughput Multiplexed Quantitation of Protein Aggregation and Cytotoxicity in a Huntington's Disease Model. *Curr Chem Genomics*, **6**, 79-86.
4. Scotter, E.L., Narayan, P., Glass, M. and Dragunow, M. (2008) High throughput quantification of mutant huntingtin aggregates. *J Neurosci Methods*, **171**, 174-179.
5. Chan, P.P. and Lowe, T.M. (2016) GtRNAdb 2.0: an expanded database of transfer RNA genes identified in complete and draft genomes. *Nucleic Acids Res*, **44**, D184-189.
6. Sherry, S.T., Ward, M.H., Kholodov, M., Baker, J., Phan, L., Smigielski, E.M. and Sirotkin, K. (2001) dbSNP: the NCBI database of genetic variation. *Nucleic Acids Res*, **29**, 308-311.
7. Schneider, C.A., Rasband, W.S. and Eliceiri, K.W. (2012) NIH Image to ImageJ: 25 years of image analysis. *Nat Methods*, **9**, 671-675.
8. Rueden, C.T., Schindelin, J., Hiner, M.C., DeZonia, B.E., Walter, A.E., Arena, E.T. and Eliceiri, K.W. (2017) ImageJ2: ImageJ for the next generation of scientific image data. *BMC Bioinformatics*, **18**, 529.
